# Accelerometry for sleep assessment in children: Criterium validity of different algorithms in wrist‐ and ankle‐worn devices

**DOI:** 10.1111/jsr.14426

**Published:** 2024-11-29

**Authors:** Pia Burger, Frea H. Kruisinga, Anneline Lettink, Mai J. M. Chinapaw, Reinoud J. B. J. Gemke

**Affiliations:** ^1^ Department of Pediatrics, Emma Children's Hospital Amsterdam UMC Amsterdam Netherlands; ^2^ Amsterdam Reproduction and Development Research Institute Amsterdam Netherlands; ^3^ Amsterdam Public Health Research Institute Amsterdam University Medical Center Amsterdam the Netherlands; ^4^ Amsterdam UMC Location Vrije Universiteit Amsterdam Public and Occupational Health Amsterdam The Netherlands

**Keywords:** accelerometry, algorithms, criterium validation, sleep

## Abstract

Polysomnography, the gold‐standard for measuring sleep, is costly, intrusive and usually limited to 1 night. Actigraphy offers a more affordable, less intrusive method over multiple nights. However, little research validates ActiGraph accelerometers against polysomnography, especially in children. This study evaluated the validity of different algorithms and compared wrist versus ankle accelerometer placements for estimating sleep in children aged 1–12 years. Twenty‐nine children undergoing overnight type 1 polysomnography wore ActiGraph accelerometers. Six algorithms were evaluated against polysomnography using Pearson correlations, intraclass correlation, paired *t*‐tests and Bland–Altman plots. Agreement was classified as poor (intraclass correlation coefficient < 0.4), fair (0.4 < intraclass correlation coefficient < 0.6), good (0.6 < intraclass correlation coefficient < 0.75) or excellent (intraclass correlation coefficient > 0.75). Total sleep time was the primary outcome. For wrist‐worn devices, the Sadeh (Actilife) and Cole–Kripke (Actilife and GGIR) algorithms showed excellent agreement with polysomnography (intraclass correlation coefficient = 0.80–0.85), while vanHees showed good agreement (intraclass correlation coefficient = 0.67) and Galland showed fair agreement (intraclass correlation coefficient = 0.46). The Cole–Kripke algorithm did not significantly differ from polysomnography total sleep time, whereas others underestimated total sleep time. For ankle‐worn devices, Sadeh (Actilife), Cole–Kripke (Actilife) and vanHees algorithms demonstrated excellent agreement (intraclass correlation coefficient = 0.75–0.82). No significant differences were found between wrist and ankle placements for certain algorithms. The findings support accelerometry as a valid tool for sleep assessment in children, recommending that algorithm selection be tailored to specific study requirements.

## 1 INTRODUCTION

Polysomnography (PSG) is the gold‐standard for measuring sleep and for detecting underlying sleep disruptors such as obstructive sleep apnea, as it can accurately characterize sleep using electroencephalography (EEG). However, PSG is limited by the number of nights that are usually evaluated (typically 1 night), its significant financial costs, and its intrusiveness. Self‐ or parent‐reported questionnaires are often used to measure children's sleep patterns, but parents are less accurate in assessing total sleep time (TST) and number of nocturnal awakenings (Sadeh, 1996). In addition, parental reports can be influenced when parent sleep is disturbed by child sleep problems.

Actigraphy offers a less intrusive and more affordable method for estimating sleep over multiple nights. This method involves accelerometer devices, typically worn on the wrist, that sense three‐dimensional wrist movements (e.g. x, y or z; Fekedulegn et al., 2020). The absence of movement is used as an indicator of sleep. However, periods of motionless wakefulness, such as reading in bed, might be misclassified as sleep. To improve the accuracy of actigraphy, self‐reported lights‐off and final wake times from sleep diaries are often used in addition to accelerometers. Currently, little research is available that addresses the validity of ActiGraph accelerometers, compared with the gold‐standard, especially regarding children (Quante et al., 2018; Smith et al., 2020). Also, only a limited number of studies have demonstrated the accuracy compared with PSG when worn at the ankle (Bélanger et al., 2013).

There are various algorithms used for estimating sleep patterns with actigraphy. The most commonly used algorithms to score sleep–wake have been developed by Sadeh et al. (1994) and Cole et al. (1992). Both algorithms were originally developed for a specific type of actigraph (AMA‐32 Motionlogger; Ambulatory Monitoring, Ardsley, NY, USA). Sadeh's algorithm was validated against a healthy sample of adolescents and young adults, whereas Cole–Kripke's algorithm was validated against an adult sample. An algorithm developed recently is the vanHees algorithm (van Hees et al., 2015), which uses accelerometer‐derived arm angle to detect sleep. The Galland algorithm has been developed in the last decade for the Actical device (Mini Mitter, Bend OR, USA), which uses a scaling process to standardize counts across the entire recording before epoch allocation, giving it the flexibility to compare different accelerometers (Galland et al., 2012a). The Galland algorithm has also been validated in Actigraph and AX3 devices (Smith et al., 2020). All algorithms are freely available as an expansion of the open‐source R‐package GGIR (Migueles et al., 2019). The Sadeh and Cole–Kripke are not exact copies of the original approach, because no complete publicly accessible description of their approach exists. However, ActiGraph offers proprietary versions of the Sadeh and Cole–Kripke algorithms through their software.

The aims of this study were to: (1) evaluate criterium validity for different algorithms (Sadeh, Cole–Kripke, vanHees and Galland) for the estimation of sleep using ActiGraph accelerometers in children aged 1–12 years; and (2) compare different site placements (wrist versus ankle) to determine the preferred placement site for measuring sleep.

## 2 METHODS

### 2.1 Participants

Participants were children (aged 1–12 years) who were scheduled for overnight PSG at a tertiary children's hospital for a clinical evaluation between April 2022 and June 2024. Patients with severe neurological conditions (e.g. tetraparesis) were excluded. All participants wore two accelerometers, one placed at the wrist and one at the ankle. The Medical Ethics Review Committee of VU University Medical Center ruled that the Medical Research involving Human Subjects Act did not apply to this study (W22_021#22.070). Informed consent was obtained for all participants from children and their parents.

### 2.2 Measures

#### 2.2.1 Polysomnography

Overnight PSG was performed with video registration using four EEG channels, bilateral electrooculography, electromyography, electrocardiography, nasal airflow measurement with nasal pressure transducer, oral airflow measurement using thermistor, measurement of end‐tidal CO_2_, transcutaneous CO_2_, chest abdominal movement and pulse oximetry. Analysis software used was BrainRT (OSG, Belgium).

Sleep recordings were first scored automatically for each 30‐s epoch, and then adjusted by a paediatrician with 12 years of experience in PSG (FK) using the American Academy of Sleep Medicine (AASM) guidelines for scoring sleep and respiratory events (Berry et al., 2012). The sleep period was scored blind from the results of the accelerometer from “lights out” to “lights on” (Table 1). The primary comparison variable of interest was TST scored as sleep between the “lights out” and “wake‐up time”. Additionally, the secondary comparison variables included wake after sleep onset (WASO), sleep efficiency (SE) and awakenings.

**TABLE 1:** Study definitions used for PSG and actigraphy.

	PSG	Actigraphy
Bedtime	Time in bed and light was switched off	
Wake‐up time	Moment in time when participant woke up for the last time before the light was switched on	
Time in bed	Minutes of time between “lights off” and “wake‐up time”	
SOL	Total time from “lights off” to first epoch period of any sleep stage	Total time from “lights off” to first epoch scored as sleep
WASO	Total number of minutes spent awake after sleep onset until wake‐up time	
TST	Total minutes of stages NREM 1–4 + REM	Total number of minutes of night‐time sleep (“time in bed” minus “WASO” minus “SOL”)
SE	TST divided by time in bed, multiplied by 100	
Awakenings	Number of paroxysms of wake activity lasting ≥ 30 s with at least 10 s of sleep preceding the change	Number of wakeful episodes lasting longer than 1 min

NREM, non‐rapid eye movement; PSG, polysomnography; REM, rapid eye movement; SE, sleep efficiency; SOL, sleep‐onset latency; TST, total sleep time; WASO, wake after sleep onset.

#### 2.2.2 Actigraphy

Participants wore two accelerometers (ActiGraph wGT3X‐BT; Pensacola, FL, USA), with nylon woven wristbands. All participants used the exact same accelerometer for the wrist and ankle. The accelerometers were initialized using ActiLife (V 6.13.4) and count data were downloaded using 10‐s epochs and the normal frequency filter. Accelerometer data were analysed using the “lights off” and “wake‐up time” information obtained from the scored PSGs. For the Sadeh (Sadeh et al., 1994) and Cole–Kripke (Cole et al., 1992) algorithms, which are embedded in Actilife, sleep estimates were calculated using the count data after aggregating to 60‐s epochs. For the Galland (Galland et al., 2012a), vanHees (van Hees et al., 2015), Sadeh and Cole–Kripke algorithms available in the open‐source R‐package GGIR version 3.1.1 raw device data were used, and epochs were aggregated to 60 s (Migueles et al., 2019). Details on how each algorithm scores an epoch as sleep versus wake are provided in Table 2. The algorithms require manual input for the bedtime and wake‐up time, which were derived from the PSG. The GGIR syntax can be found in Supplementary Information S1. The computers performing the PSG and the actigraphy were synchronized to standard time.

**TABLE 2:** Explanation of how each algorithm scores an epoch as sleep or wake.

Algorithm	Explanation
Sadeh (ActiLife) (Actigraph, 2018; Sadeh et al., 1994)	The algorithm uses the *y*‐axis epoch data and limits any counts > 300 to a maximum value of 300 Uses an 11‐min window (1‐min epoch and the preceding and following 5‐min) to compute the probability of sleep (PS) of the current/scored epoch as follows: $\mathrm{PS}=7.601–0.065\;\text{Mean}-W-5\;\;\min–1.08\;\mathrm{NAT}–0.56\;\mathrm{SD}-\text{last}\;6\;\min–0.073\;\ln\;\left(\mathrm{ACT}\right)$ where Mean − *W* − 5 min is the average number of activity counts during the scored epoch and the window of five epochs preceding and following it; SD – last 6 min is the standard deviation of the activity counts during the scored epoch and the five epochs preceding it; NAT is the number of epochs with activity counts ≥ 50 and < 100 counts in the 11‐min window; ln (ACT) is the natural logarithm of the activity counts during the scored epoch A specific epoch is scored as sleep if PS is > −4
Sadeh (GGIR) (Migueles et al., 2019)	Similar to Sadeh (ActiLife), but algorithm uses an implementation of the zero‐crossing counts and a specific epoch is considered as sleep if PS ≥ 0 To use the GGIR implementation of the zero‐crossing counts and Sadeh algorithm, specify argument HASIB.algo = “Sadeh1994” and argument Sadeh_axis = “Y” to indicate that the algorithm should use the *y*‐axis of the sensor
Cole–Kripke (Actigraph, 2018; Cole et al., 1992) (ActiLife)	The algorithm uses *y*‐axis epoch data, divides the activity counts by 100, and limits any scaled counts > 300 to a maximum value of 300 Uses a 7‐min window (1‐min epoch, the preceding 4‐min and the following 2‐min) and is computed as follows: $S=0.001\;\left(106\;A_{-4}+54\;A_{-3}+58\;A_{-2}+76\;A_{-1}+23\;A_{0}+74\;A_{1}+67\;A_{2}\right),$ where S is the weighted sum of the activity counts and *A*_*n*_ are the activity counts for the corresponding minute *n*, counted from the scored epoch The current epoch is scored as sleep when *S* < 1; if *S* ≥ 1 it is scored as wake (Actigraph, 2018) It includes the Webster rescoring rules: (a) after at least 4 min scored as wake, the next 1 min scored as sleep is rescored as wake; (b) after at least 10 min scored as wake, the next 3 min scored as sleep are rescored as wake; (c) after at least 15 min scored as wake, the next 4 min scored as sleep are rescored as wake; (d) 6 min or less scored as sleep surrounded by at least 10 min (before and after) scored as wake are rescored as wake
Cole–Kripke (GGIR) (Migueles et al., 2019)	Similar to Cole–Kripke (ActiLife), but the algorithm uses an implementation of the zero‐crossing counts and skips the rescoring rules as the original paper showed marginal added value of this added complexity To use the GGIR implementation, specify argument HASIB.algo = “ColeKripke1992”, Sadeh_axis = “Y”
Gallland (Galland et al., 2012b)	This algorithm is like the Cole–Kripke algorithm, with the exception that it is performed using count‐scaled data Each epoch is scaled relative to the mean value of all epochs that have non‐zero counts Thus, dividing each epoch activity count by the mean activity count of the whole recording Once this stage has been completed, the algorithm operates in a similar way to the Cole–Kripke algorithm The Galland (also called count‐scaled) algorithm uses a window of 105 s (the current 15‐s epoch, the preceding four epochs and the following two epochs), and is computed as follows: $S=2.7\;\left(1.17\;W_{-4}+1.09\;W_{-3}+2.57\;W_{-2}+4.30\;W_{-1}+5.05\;W_{0}+4.01\;W_{1}+0.82\;W_{2}\right),$ where *S* is the scaled sum of the activity scores, and *W*_*n*_ are the activity counts for the corresponding epoch *n*, counted from the scored epoch The current epoch is scored as sleep when *S* < 1; if *S* ≥ 1 it is scored as wake To use the GGIR implementation, specify argument HASIB.algo = “Galland2012”, Sadeh_axis = “Y”,
vanHees (Migueles et al., 2019; van Hees et al., 2015)	Uses accelerometer‐derived arm angle to detect sleep (as opposed to acceleration like the other algorithms) Arm angle is estimated as follows: ${\text{angle}}_{z}=\tan^{-1}\frac{a_{z}}{\sqrt{a_{x}^{\;2}+a_{y}^{\;2}}}\times 180/\pi$ where *a*_*x*_, *a*_*y*_ and *a*_*z*_ are the median values of the three orthogonally positioned raw acceleration sensors in g‐units based on a rolling 5‐s time window The *z*‐axis corresponds to the dorsal–ventral direction when the wrist is in the anatomical position Estimated angles are averaged per 5‐s epoch and are used to assess changes in arm angle between successive 5‐s epochs Periods of time during which there are no changes > 5° over at least 5 min are classified as bouts of sustained inactivity (sleep periods)

### 2.3 Power calculation

Sample size was based on expected differences in TST as our primary outcome variable to compare accelerometry with PSG as the gold‐standard. Based on a mean TST of 660 ± 38 min in children 3–7 years old (Carter et al., 2011), at least 22 subjects were required to detect a (clinically relevant) difference of 30 min at the 5% level of significance and a power of 90%.

### 2.4 Statistical analysis

Data analyses were performed in R (version 4.4.0). Descriptive statistics are presented as mean ± SD for continuous variables and count (%) for categorical variables. Comparisons between algorithms and PSG were assessed using Pearson correlation coefficients, paired *t*‐tests, intraclass correlation coefficients (ICC; method: two‐way fixed‐effects single measures), box and whisker plots, and Bland–Altman plots. Paired *t*‐tests were also conducted for the comparison of accelerometer placements. For the paired *t*‐tests, a significance level of < 0.001 was set to control for multiple pairwise comparisons. Agreement was considered poor if the ICC was below 0.4, fair if the ICC values were between 0.40 and 0.59, good if the ICC values were between 0.6 and 0.74, and excellent if the ICC was above 0.75 (Hallgren, 2012). The Bland–Altman plots present the difference between accelerometer algorithms and PSG on the *y*‐axis and the mean PSG value on the *x*‐axis for each sleep parameter, and show a fitted regression line (Krouwer, 2008).

## 3 RESULTS

**FIGURE 1:**
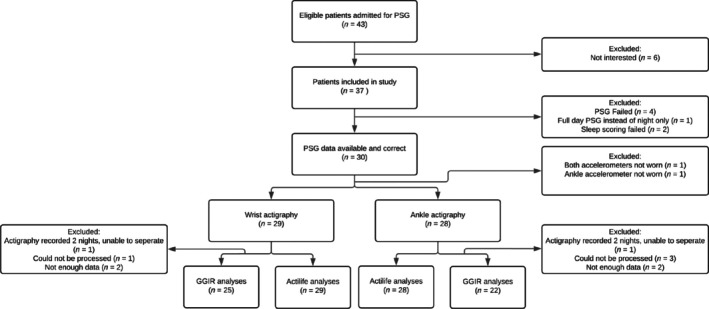
Flow diagram eligible, included and analysed participants.

Twenty‐nine participants were included in the analysis (Figure 1). The sample consisted of slightly more boys (62%) than girls, who were on average 5.6 (Quante et al., 2018) years old. A total of 24 participants (83%) underwent PSG for suspicion of obstructive sleep apnea syndrome (OSAS), three (10%) for hypoventilation syndrome, and two (7%) for other reasons.

**FIGURE 2:**
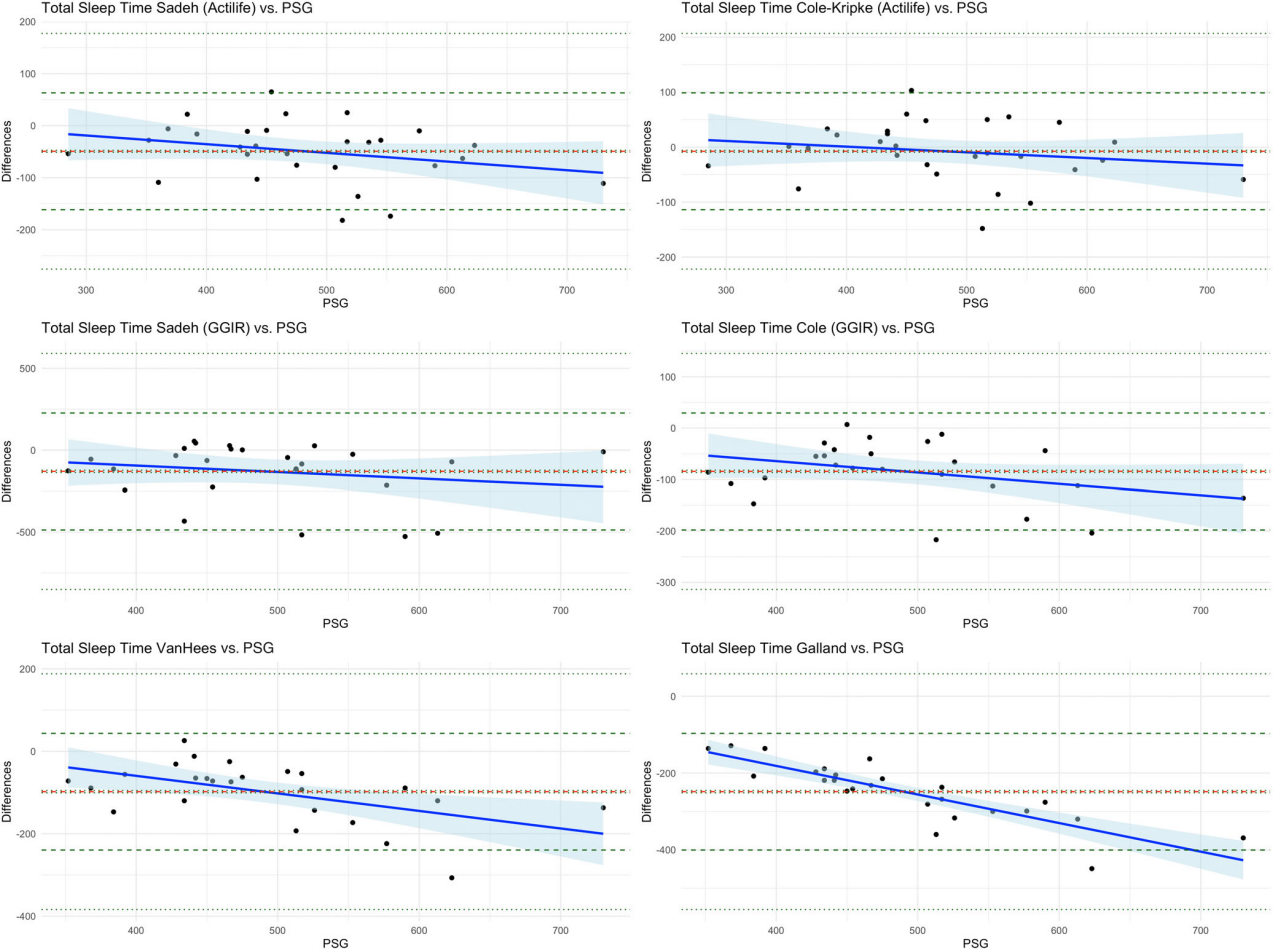
Bland–Altman plots of wrist‐worn actigraphy analysed with six different algorithms and polysomnography (PSG) measurement of total sleep time (TST) on the same night (*n* = 29). The *x*‐ and *y*‐axes in minutes. With regression slope (blue ‐‐‐‐) and 95% confidence interval (CI; shaded blue), mean difference (red ‐ ‐ ‐), and upper and lower limits of agreement (green ‐ ‐ ‐).

Moderate correlations were found between PSG TST and wrist‐worn actigraphy TST for the following algorithms (Figure S2.1): Sadeh (Actilife, *r* = 0.82); Cole–Kripke (Actilife, *r* = 0.85); and vanHees (*r* = 0.64). The Cole–Kripke (Actilife) algorithm did not show a significant difference with PSG TST (mean difference = −8 min, 95% confidence interval [CI] [−28, 13]) and the ICC was 0.85, indicating excellent agreement (Table 3). The Cole–Kripke (GGIR) also showed excellent agreement, but TST was significantly underestimated (mean difference = −84 min, 95% CI [−108, 60]). The Sadeh (Actilife) algorithm showed an ICC of 0.82 (excellent agreement), yet TST was significantly underestimated (mean difference = −49 min, 95% CI [−71, −27]). The vanHees algorithm showed good agreement (ICC = 0.67) but also showed a significant underestimation (mean difference = −98 min, 95% CI [−127, −68]). The Galland algorithm showed fair agreement, yet underestimated TST significantly (mean difference = −248 min, 95% CI [−280, −216]; see Figure S2.2 for the corresponding boxplots). The Bland–Altman plots are shown in Figure 2. The regression slope for the Galland algorithm indicated significant proportional bias (*r* = −0.74), with greater overestimation of TST by actigraphy at shorter PSG TST.

**TABLE 3:** Comparison of actigraphy algorithms and placements with PSG sleep parameters.

Sleep parameter	Algorithm	Placement	Mean (SD)	Mean Δ (95% CI) act‐PSG	ICC	Mean Δ (95% CI) ankle‐wrist
TST (min)	PSG	PSG	482 (95)			
	Sadeh (Actilife)	Wrist	433 (96)	−49 (−71, −27)*	0.82	47 (24, 69)*
		Ankle	476 (104)	−7 (−35, 21)	0.75	
	Cole–Kripke (Actilife)	Wrist	458 (100)	−8 (−28, 13)	0.85	54 (34, 76)*
		Ankle	548 (100)	43 (20, 66)*	0.82	
	Sadeh (GGIR)	Wrist	360 (186)	−130 (−205, −55)*	0.22	11 (−65, 88)
		Ankle	369 (191)	−112 (−196, −29)	0.20	
	Cole–Kripke (GGIR)	Wrist	406 (88)	−84 (−108, −60)*	0.80	−22 (−69, 25)
		Ankle	385 (118)	−97 (−141, −54)*	0.57	
	vanHees	Wrist	392 (79)	−98 (−127, −68)*	0.67	−6 (−23, 12)
		Ankle	392 (79)	−89 (−116, −63)*	0.78	
	Galland	Wrist	241 (46)	−248 (−280, −216)*	0.46	−3 (−20, 14)
		Ankle	241 (56)	−240 (−274, −206)*	0.53	
WASO (min)	PSG	PSG	74 (51)			
	Sadeh (Actilife)	Wrist	128 (63)	54 (32, 76)*	0.50	−37 (−56, −17)*
		Ankle	89 (63)	20 (−4, 46)	0.26	
	Cole–Kripke (Actilife)	Wrist	91 (56)	18 (−2, 37)	0.53	−46 (−65, −26)*
		Ankle	44 (40)	−24 (−42, −5)	0.33	
	Sadeh (GGIR)	Wrist	53 (112)	−28 (−79, 23)	0.01	−2 (−85, 82)
		Ankle	54 (134)	−20 (−87, 46)	0.00	
	Cole–Kripke (GGIR)	Wrist	123 (47)	42 (19, 64)*	0.42	−2 (−26, 21)
		Ankle	120 (49)	45 (18, 73)	0.09	
	vanHees	Wrist	124 (51)	43 (19, 67)	0.34	−2 (−21, 17)
		Ankle	120 (56)	46 (17, 74)*	0.15	
	Galland	Wrist	282 (74)	201 (166, 2437)*	0.13	3 (−14, 21)
		Ankle	284 (75)	209 (174, 245)*	0.15	
SE (%)	PSG	PSG	82 (10)			
	Sadeh (Actilife)	Wrist	75 (10)	−7 (−11, −3)	0.42	8 (4, 12)*
		Ankle	83 (11)	0 (−6, 5)	0.03	
	Cole–Kripke (Actilife)	Wrist	82 (10)	0 (−4, 4)	0.43	9 (5, 13)*
		Ankle	92 (7)	8 (3, 13)	0.02	
	Sadeh (GGIR)	Wrist	64 (29)	−17 (−29, −5)	0.10	2 (−12, 16)
		Ankle	67 (28)	−15 (−28, −2)	0.07	
	Cole–Kripke (GGIR)	Wrist	68 (11)	−14 (−18, −11)*	0.53	0 (−3, 3)
		Ankle	70 (10)	−12 (−17, −8)*	0.48	
	vanHees	Wrist	66 (11)	−15 (−20, −10)*	0.33	−1 (−4, 2)
		Ankle	68 (10)	−14 (−20, −9)*	0.26	
	Galland	Wrist	41 (8)	−40 (−45, −35)*	0.18	−1 (−4, 3)
		Ankle	42 (8)	−40 (−46, −34)*	0.00	
Awakenings (*n*)	PSG	PSG	20 (10)			
	Sadeh (Actilife)	Wrist	20 (8)	0 (−4, 4)	0.38	−3 (−6, −1)
		Ankle	17 (9)	0 (−3, 5)	0.24	
	Cole–Kripke (Actilife)	Wrist	20 (8)	0 (−4, 4)	0.18	−8 (−11, −5)*
		Ankle	12 (8)	−7 (−12, −3)	0.27	
	Sadeh (GGIR)	Wrist	4 (3)	−17 (−21, −12)*	0.00	0 (−1, 2)
		Ankle	4 (3)	−16 (−21, −12)*	0.00	
	Cole–Kripke (GGIR)	Wrist	24 (10)	4 (−2, 9)	0.17	−1 (−4, 3)
		Ankle	23 (10)	3 (−2, 8)	0.42	
	vanHees	Wrist	17 (5)	−3 (−7, 1)	0.15	−1 (−2, 1)
		Ankle	17 (5)	−3 (−8, 1)	0.20	
	Galland	Wrist	23 (8)	2 (−3, 7)	0.00	−2 (−4, 0)
		Ankle	21 (8)	1 (−4, 6)	0.09	

Comparisons were made using paired *t*‐tests

* Behind mean differences indicate a *p*‐value below 0.001. CI, confidence interval; ICC, intraclass correlation coefficient; PSG, polysomnography; SE, sleep efficiency; TST, total sleep time; WASO, wake after sleep onset.

Moderate correlations between PSG TST and ankle‐worn actigraphy TST were found for Sadeh (Actilife, *r* = 0.74), Cole–Kripke (Actilife, *r* = 0.82) and vanHees (*r* = 0.74; Figure S2.3). Sadeh (Actilife), Cole–Kripke (Actilife) and vanHees showed excellent agreement (ICC = 0.75, 0.82 and 0.78, respectively). The Sadeh (Actilife) algorithm did not show a significant difference compared with PSG (mean difference = −7 min, 95% CI [−35, 21]). However, Cole–Kripke (Actilife) showed a significant overestimation (mean difference = 43 min, 95% CI [20, 66]). The Galland algorithm showed a fair agreement, with a significant underestimation (mean difference = −240 min, 95% CI [−274, −206]; see Figure S2.4 for the corresponding boxplots). The Bland–Altman plots (Figure S2.5) showed similar results to those with wrist actigraphy measurements. The difference between wrist and ankle placements was only significant for the Sadeh (Actilife) and Cole–Kripke (Actilife) algorithms.

For WASO, SE and awakenings, none of the algorithms showed a significant correlation with PSG sleep parameters. Wrist‐worn accelerometry had fair agreement with PSG regarding WASO and SE for the Sadeh (Actilife) and Cole–Kripke (Actilife, and GGIR), while the other algorithms and placements had poor agreement. All the algorithms, regardless of placement, showed poor agreement for number of awakenings. For WASO, there was no significant difference for wrist‐worn accelerometry for the Cole–Kripke (Actilife; mean difference = 18 min, 95% CI [−2, 37]), Sadeh (GGIR; mean difference = −28 min, 95% CI [−79, 23]) and vanHees (mean difference = 43 min, 95% CI [19, 64]) algorithms, while for ankle‐worn accelerometry no difference was found for Sadeh (Actilife and GGIR) and Cole–Kripke (Actilife and GGIR). Correlation, box‐ and Bland–Altman plots are presented in Figures S3.6–S5.24.

## 4 DISCUSSION

The aim of this study was to evaluate criterion validity of different algorithms (Sadeh, Cole–Kripke, vanHees and Galland) for the estimation of sleep using ActiGraph GT3X+ accelerometers in children aged 1–12 years, and to compare different site placements (wrist versus ankle) to determine the best site for measuring sleep. We found that the Cole–Kripke (Actilife) scoring algorithm had stronger agreement compared with the other algorithms with wrist‐worn actigraphy, and showed no statistical differences compared with PSG for any of the sleep parameters. The Cole–Kripke (GGIR) and Sadeh (Actilife) algorithms also showed excellent agreement with PSG TST for wrist‐worn actigraphy, yet the mean differences were more pronounced and significantly different from PSG TST (mean difference = −84 min, 95% CI [−108, 60] and −49 min, 95% CI [−71, −27], respectively). The other algorithms showed fair to good agreement, with significant differences between wrist‐worn actigraphy and PSG TST. These findings correspond to previous studies. Earlier research showed excellent agreement between ActiGraph GT3X+ accelerometers worn at the wrist and PSG TST (ICC = 0.79), yet also showed an underestimation of TST (mean difference = −48 min, 95% CI [−97, −0.5]) and an overestimation of WASO (mean difference = 60 min [95% CI 36, 30]) in adolescents when using the Sadeh (Actilife; Quante et al., 2018). They also showed that the Cole–Kripke (Actilife) had higher agreement with PSG, and a smaller mean difference of −18.2 min, 95% CI [67, 30] (Quante et al., 2018). Other research examined the Sadeh and Cole–Kripke algorithms in children aged 3–18 years wearing the Motionlogger Sleep Watch compared with PSG. They found that the Sadeh algorithm underestimated TST by 24 min, 95% CI [−31, −16], and the Cole–Kripke algorithm only by 2.4 min, 95% CI [−9, 4] (Meltzer et al., 2012).

As far as we know, there are currently no papers published about the validation of any of our included algorithms for ankle‐worn (wGT3X‐BT) actigraphy. Especially in younger children, who might not tolerate actigraphy worn on the wrist, wearing it on the ankle could be a viable option if proven valid. For ankle‐worn actigraphy, the Cole–Kripke algorithm showed the highest agreement with PSG for estimating TST (ICC = 0.82), yet it significantly overestimated TST (mean difference = 43 min, 95% CI [20, 66]). Similarly, the vanHees algorithm showed excellent agreement for TST when compared with PSG, yet TST was significantly underestimated (mean difference = −89 min, 95% CI [−116, −63]). The Sadeh (Actilife) also showed excellent agreement regarding TST. Our study did not find any significant difference for any of the sleep parameters between Sadeh (Actilife) and PSG for ankle‐worn actigraphy. There is limited research about validating ankle‐worn actigraphy in children. Two studies using the Actiwatch‐L (Mini‐Mitter) found no significant difference in TST compared with PSG when worn on the ankle in children aged 2–5 years (Bélanger et al., 2013; Sitnick et al., 2008).

When comparing wrist and ankle placements, the Sadeh (Actilife) and Cole–Kripke (Actilife) algorithms showed significant differences for all sleep parameters, while the other algorithms showed none. Studies investigating wrist and ankle placement in children between 2 and 5 years old using an Actiwatch‐L (Mini‐Mitter) found no statistical differences in sleep parameters (using different algorithms; Bélanger et al., 2013). However, our study showed that the Sadeh (Actilife) and Cole–Kripke (Actilife) algorithms overestimated TST and underestimated WASO when the accelerometer was worn at the ankle. The algorithms implemented in GGIR did not show differences, indicating that the placement of the accelerometer is interchangeable. This could help include young children in research, as they might easily drop‐out if the only option provided was wrist‐worn actigraphy.

The Galland algorithm performed poorer compared with the other algorithms. With both wrist and ankle placements, it showed fair agreement with PSG TST, while underestimating TST with a mean difference of approximately −248 min (95% CI [−280, −216]). Additionally, the Bland–Altman plots showed proportional bias for all sleep parameters. Earlier research in children aged 5–8 years showed a mean difference of −26 min (95% CI [−29, 3]) for the ActiGraph GT3X+ accelerometer worn at the wrist using the Galland algorithm (Smith et al., 2020). Similarly, recent research conducted in children between 8 and 16 years old showed an overestimation of the Galland algorithm of only 14 min (95% CI [−1, 29]) when worn at the wrist, where it was processed in Python (Meredith‐Jones et al., 2024). The difference might be explained by their use of the algorithm for processing in Matlab, which is how it was originally developed (Galland et al., 2012a). However, as far as we know, the mathematical equations remained the same. The vanHees algorithm also showed a significant underestimation of TST (about 95 min for both wrist‐ and ankle‐worn actigraphy) and SE (15% for both site placements). However, it showed good agreement (wrist‐worn ICC = 0.68, ankle‐worn ICC = 0.78) with PSG TST, which is higher compared with earlier research in an adult population using wrist‐worn actigraphy analysed with the vanHees algorithm (ICC = 0.39; Sansom et al., 2023).

This study has several strengths. The inclusion of children between 1 and 12 years old allowed us to examine validity in both pre‐schoolers and schoolchildren. The simultaneous placement of two accelerometers (wrist and ankle) allowed us to assess the validity of the wGT3XBT placed at two sites as compared with the gold‐standard. The comparison of multiple scoring algorithms, both open‐source and proprietary, enabled us to determine the best algorithm for sleep scoring in children. Finally, as using only one statistical approach (e.g. correlation, paired *t*‐test) can provide misleading agreement between devices, multiple statistical approaches were employed in this study, including Pearson correlations, boxplots, paired *t*‐tests and Bland–Altman plots, all with correction for multiple testing. There are also some limitations. Although our sample size in (young) children was larger compared with other studies and met the minimum required to detect significant differences, it is still relatively small. Additionally, TST varies across ages and our sample size was calculated based on children aged 3–7 years; however, other studies included similar numbers of participants for similar or broader age ranges (Esbensen, 2016; Sadaka et al., 2014). Additionally, the participants were a clinical sample with issues such as OSAS and hypoventilation, so extrapolating our results to a healthy population should be done with caution.

## 5 CONCLUSION

Our study indicates that the wrist‐worn ActiGraph wGT3XBT accelerometer is a valid device for estimating various sleep parameters. We showed that multiple algorithms are appropriate to use; however, it is important to note that TST is underestimated by all algorithms. Additionally, if wrist‐worn actigraphy is less suitable (e.g. in younger children), ankle‐worn actigraphy may be used as an alternative. If interchangeability of placements is important, the algorithms implemented in GGIR might be best to use. These findings support the use of wGT3XBT actigraphy as a valid tool for sleep assessment in both preschool and school‐aged children, providing flexibility in algorithm selection based on the specific requirements and circumstances of the study population.

## AUTHOR CONTRIBUTIONS

**Pia Burger:** Conceptualization; methodology; software; data curation; investigation; formal analysis; project administration; writing – original draft; writing – review and editing. **Frea H. Kruisinga:** Investigation; writing – review and editing. **Anneline Lettink:** Methodology; writing – review and editing. **Mai J. M. Chinapaw:** Writing – review and editing. **Reinoud J. B. J. Gemke:** Writing – review and editing; conceptualization; methodology; supervision; investigation.

## FUNDING INFORMATION

None.

## CONFLICT OF INTEREST STATEMENT

None.

## Supporting information


**DATA S1.** Supporting Information.

## Data Availability

Research data are not shared.
