# Supplementary material for: Accelerometry for sleep assessment in children: Criterium validity of different algorithms in wrist‐ and ankle‐worn devices
Source: J Sleep Res. 2024 Nov 29;34(4):e14426. doi: 10.1111/jsr.14426 (PMC12215244; doi:10.1111/jsr.14426)
Supplement: Supplementary file 1 — DATA S1. Supporting Information. [file JSR-34-e14426-s001.pdf]

Accelerometry for sleep assessment in children: criterium validity of different algorithms in wrist- and ankle-worn devices – **Supplementary Information**

Pia Burger[1.2,3], Frea H. Kruisinga[1], Anneline Lettink[3,4], Mai J.M. Chinapaw[3,4], Reinoud J.B.J. Gemke[1,2,3]

[1] Department of Pediatrics, Emma Children's Hospital, Amsterdam UMC, Amsterdam, Netherlands.

[2] Amsterdam Reproduction and Development research institute, Amsterdam, Netherlands.

[3] Amsterdam Public Health Research Institute, Amsterdam University Medical Center, , Amsterdam, the Netherlands

[4] Amsterdam UMC Location Vrije Universiteit Amsterdam, Public and Occupational Health, Amsterdam, The Netherlands

Table of contents

S1. GGIR syntax......3

S2. Analysis Total Sleep Time......5

S3. Analysis Wake after sleep onset......10

S4. Analysis sleep efficiency......16

S5. Analysis awakenings......22

S1. GGIR syntax

```
library(GGIR)

GGIR(datadir=" Path ",
    outputdir="Path ",
    do.report=c(2,4,5),
     #=====================
     # Part 2
     #=====================
     data_masking_strategy = 1,
     hrs.del.start = 0,         hrs.del.end = 0,
     maxdur = 9,                includedaycrit = 16,
     qwindow=c(0,24),
     mvpathreshold =c(100),
     excludefirstlast = FALSE,
     includenightcrit = 16,
     epochvalues2csv = FALSE,
     #=====================
     # Part 3 + 4
     #=====================
           loglocation= "Path",
           outliers.only = TRUE,
           sleepwindowType = "TimeInBed",
           criterror = 4,
           relyonsleeplog = TRUE,
           sleeplogidnum = TRUE,
           colid=1,
           coln1=2,
           do.visual = TRUE,
           nnights = 1,
     #=====================
     # Part 5
     #=====================
     threshold.lig = c(30), threshold.mod = c(100),  threshold.vig = c(400),
     boutcriter = 0.8,      boutcriter.in = 0.9,     boutcriter.lig = 0.8,
     boutcriter.mvpa = 0.8, boutdur.in = c(1,10,30), boutdur.lig = c(1,10),
     boutdur.mvpa = c(1),
     includedaycrit.part5 = 2/3,
     save_ms5rawlevels = TRUE,
     save_ms5raw_without_invalid = FALSE,
     part5_agg2_60seconds=TRUE,
     #=====================
     # Visual report
     #=====================
     timewindow = c("WW"),
     visualreport=TRUE)
```

For Galland, add:

```
HASIB.algo = "Galland2012",
        Sadeh_axis = "Y",
```

For Sadeh, add:

```
HASIB.algo = "Sadeh1994",
        Sadeh_axis = "Y",
```

For Cole-Kripke, add:

```
HASIB.algo = "ColeKripke1992",
        Sadeh_axis = "Y",
```

S2. Analysis Total Sleep Time

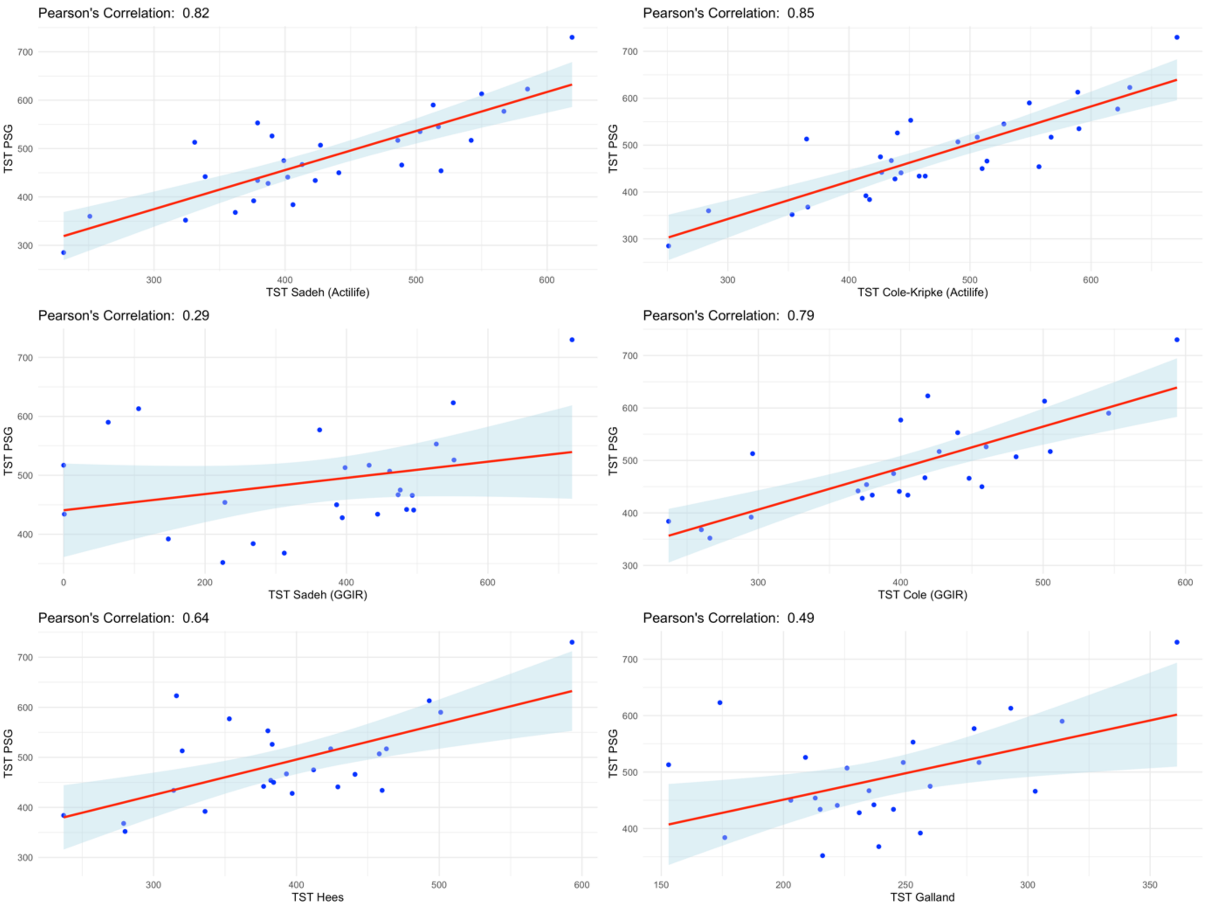

Figure 1 Correlation of wrist-worn actigraphy analyzed with 6 different algorithms and polysomnography (PSG) measurement of total sleep time (TST) on the same night. Axes are in minutes. With regression slope (red) and 95% confidence interval (shaded blue).

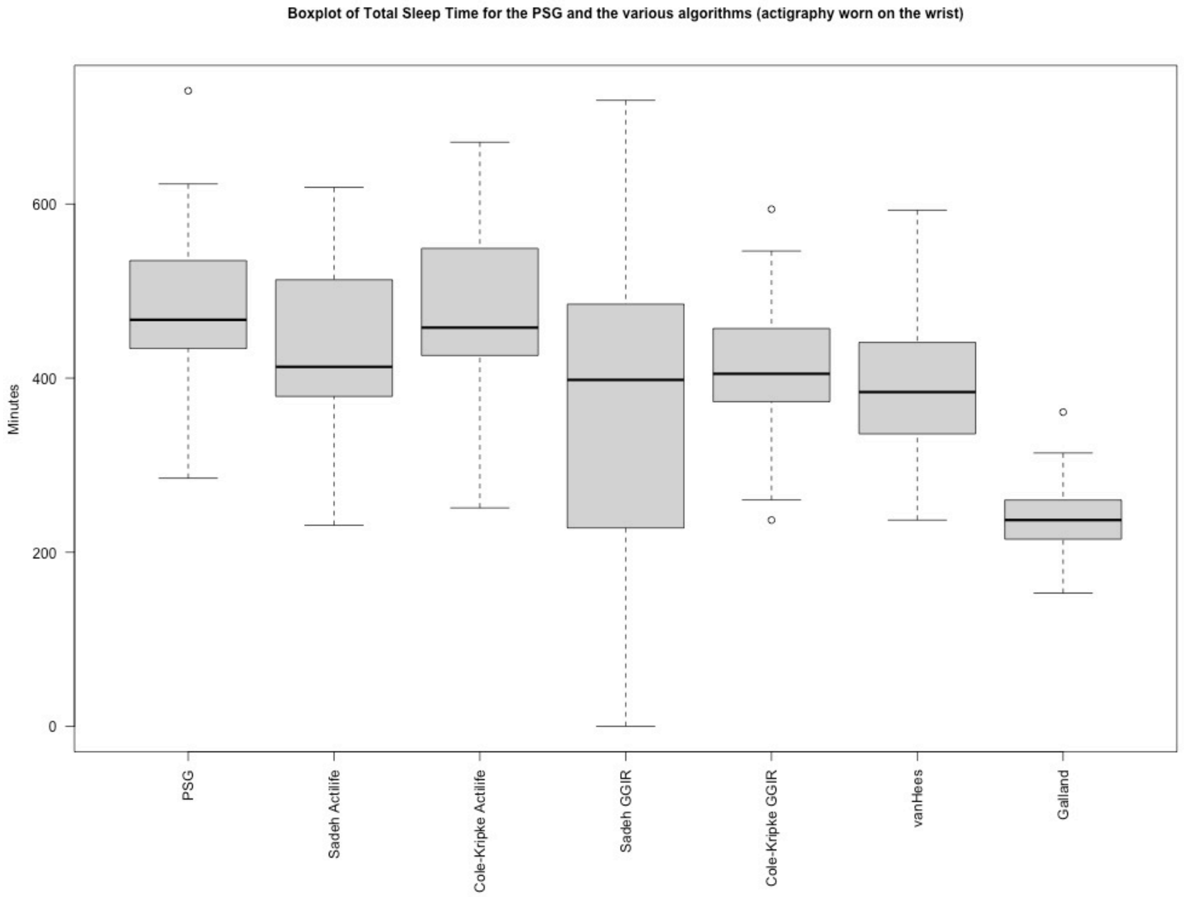

Figure 2 Boxplot (median, first and third quartiles, and range) of TST measured from wrist actigraphy and PSG.

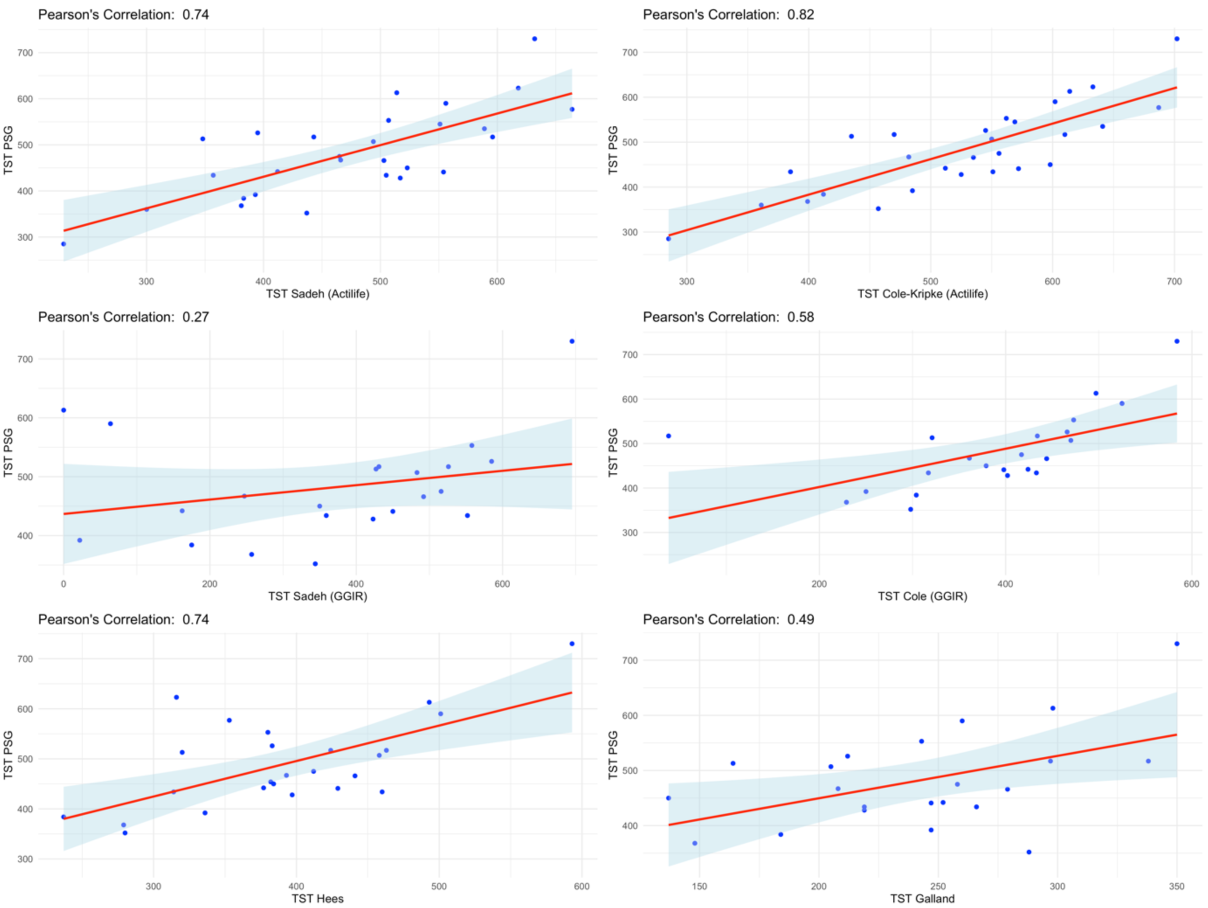

Figure 3 Correlation of ankle-worn actigraphy analyzed with 6 different algorithms and polysomnography (PSG) measurement of total sleep time (TST) on the same night. Axes are in minutes. With regression slope (red) and 95% confidence interval (shaded blue).

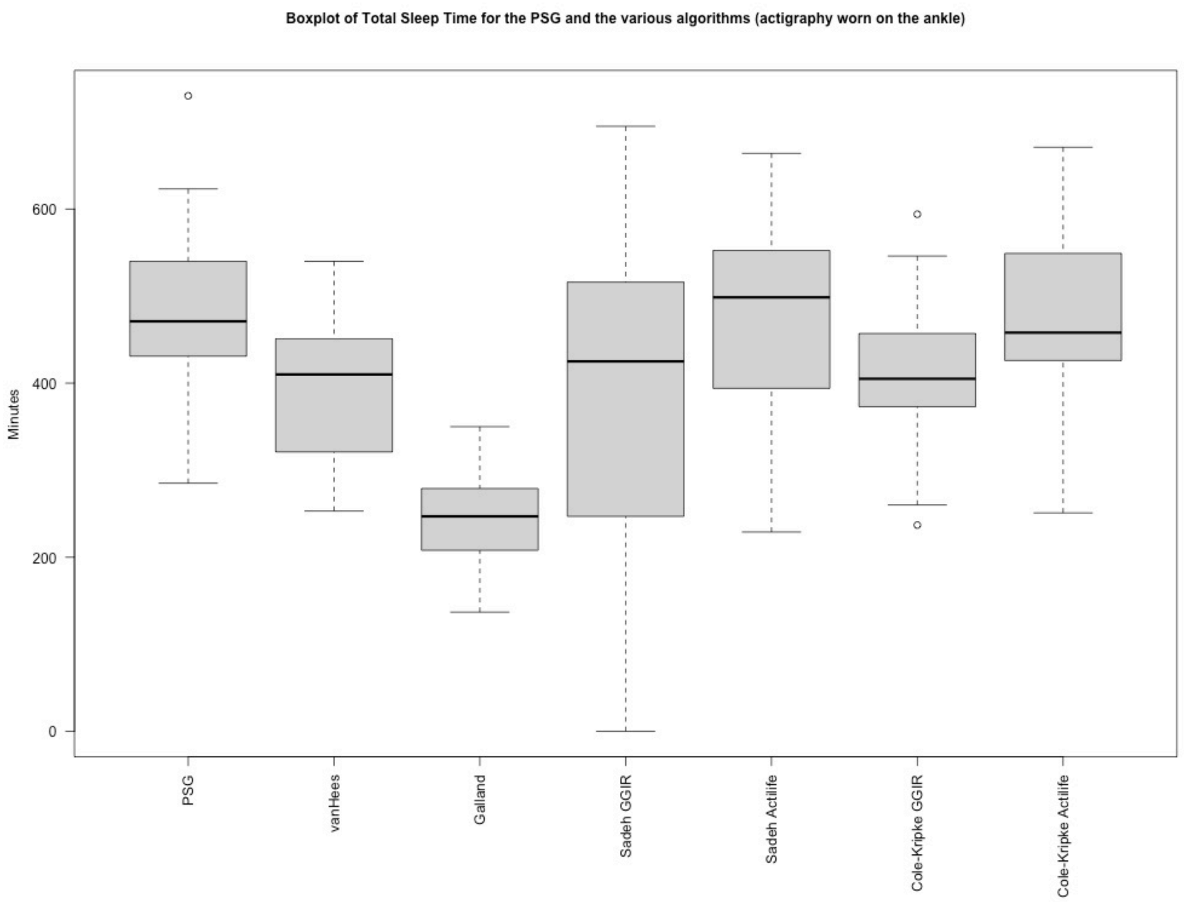

Figure 4 Boxplot (median, first and third quartiles, and range) of TST measured from ankle actigraphy and PSG.

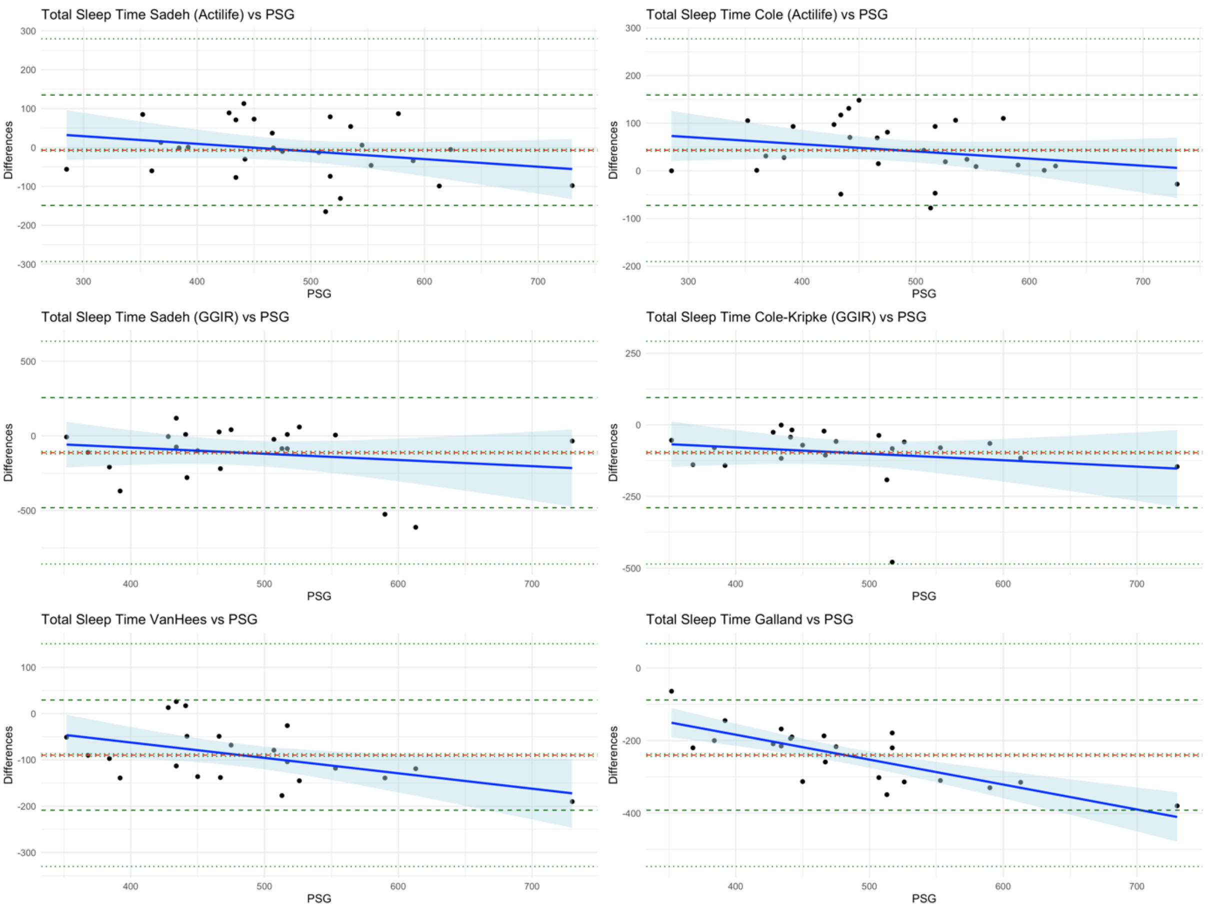

Figure 5 Bland-Altman plots of ankle-worn actigraphy analyzed with 6 different algorithms and polysomnography (PSG) measurement of total sleep time (TST) on the same night. Axes are in minutes. The Galland algorithm (r=-0.69) showed significant proportional bias. With regression slope (blue ----) and 95% confidence interval (shaded blue), mean difference (red - - -), and upper and lower limits of agreement (green - - -).

S3. Analysis Wake after sleep onset

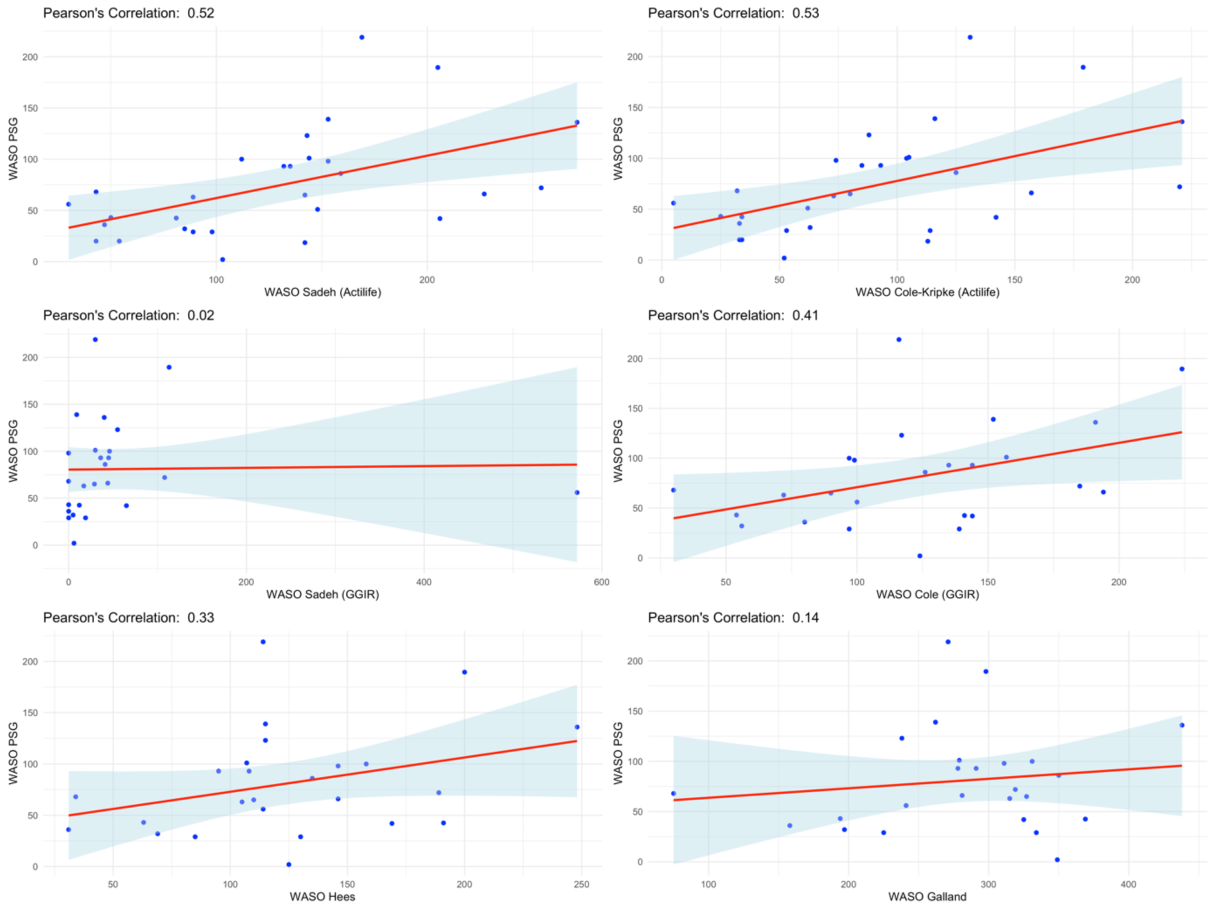

Figure 6 Correlation of wrist-worn actigraphy analyzed with 6 different algorithms and polysomnography (PSG) measurement of Wake after sleep onset (WASO). Axes are in minutes. None of the algorithms were significantly related to PSG WASO. With regression slope (red) and 95% confidence interval (shaded blue).

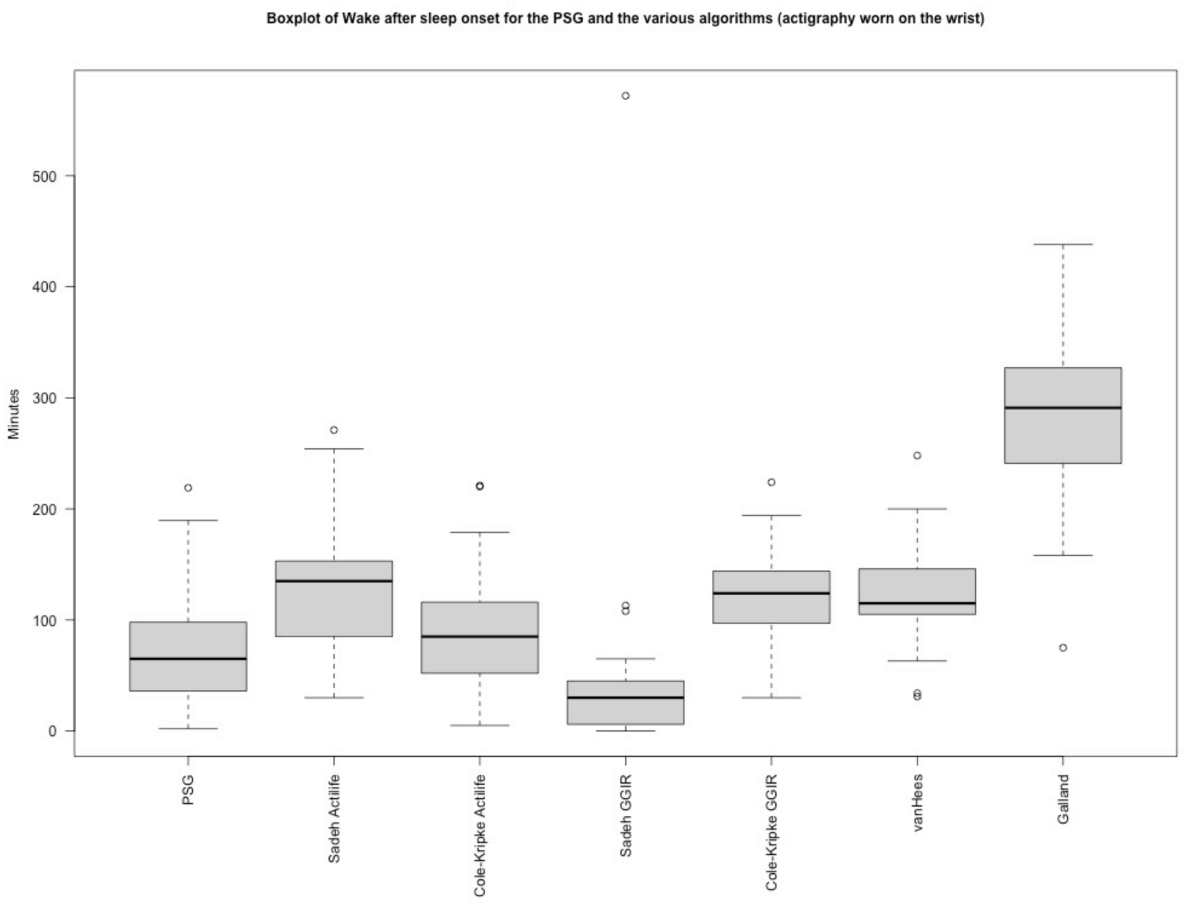

Figure 7 Boxplot (median, first and third quartiles, and range) of Wake after sleep onset measured from wrist actigraphy and PSG.

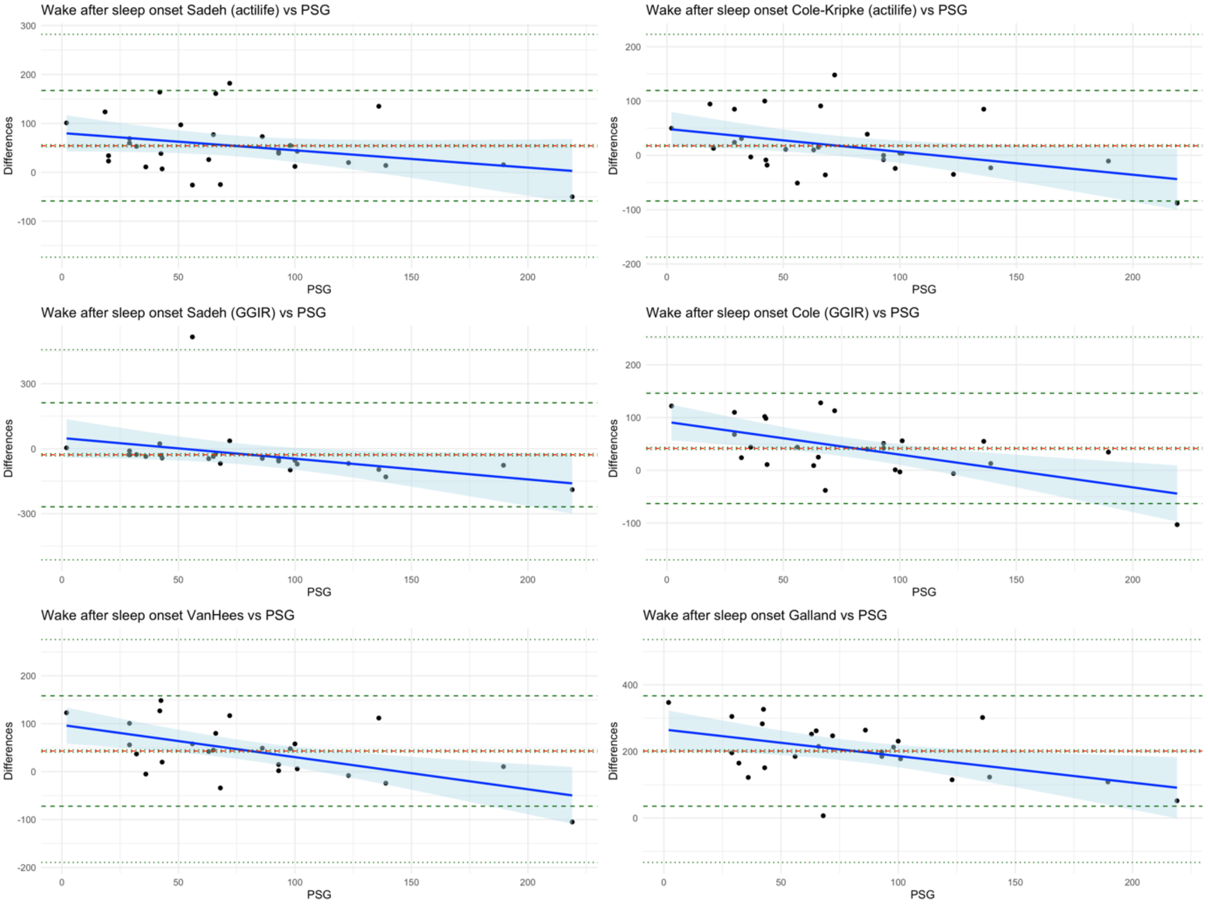

Figure 8 Bland-Altman plots of wrist-worn actigraphy analyzed with 6 different algorithms and polysomnography (PSG) measurement of Wake after sleep onset on the same night. Axes are in minutes. None of the algorithms showed significant proportional bias. With regression slope (blue ----) and 95% confidence interval (shaded blue), mean difference (red - - -), and upper and lower limits of agreement (green - - -).

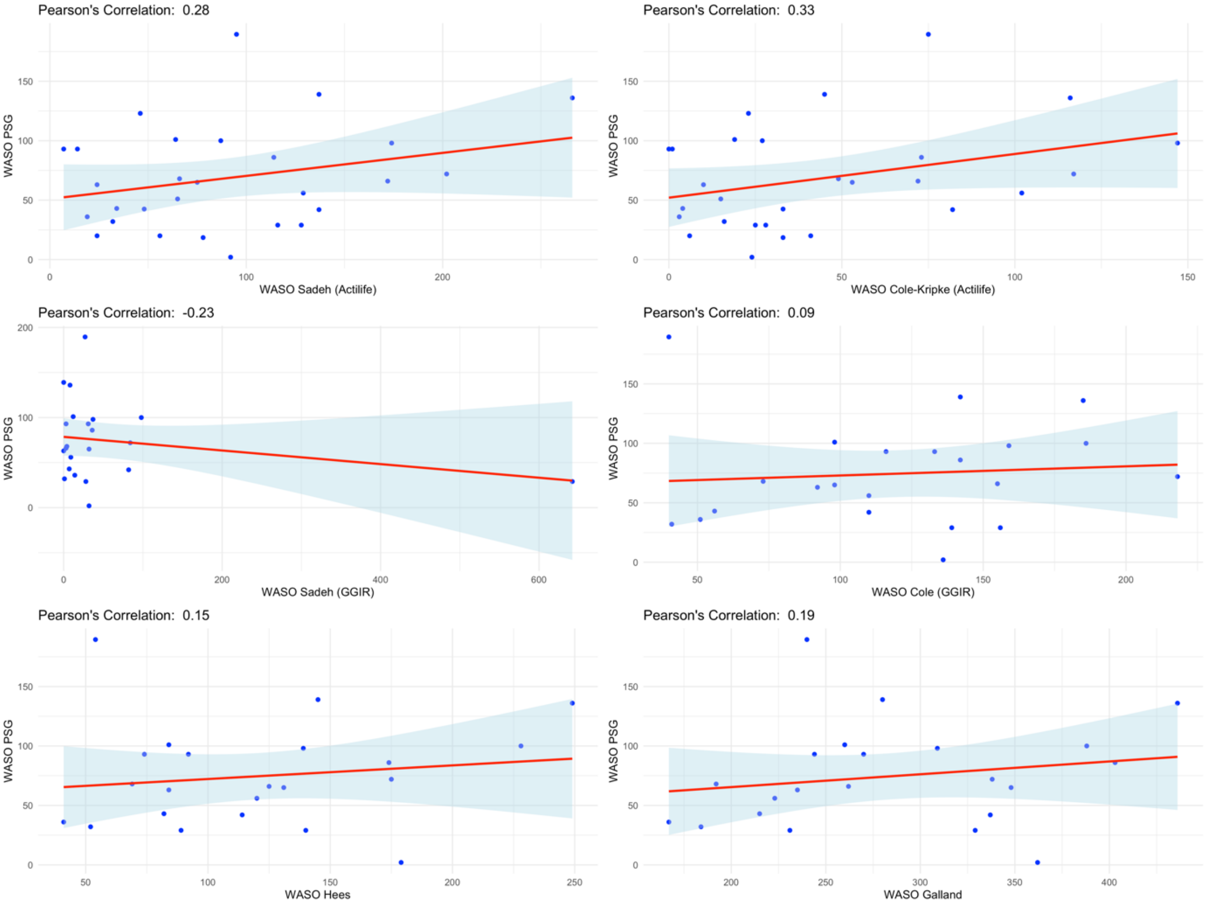

Figure 9 Correlation of ankle-worn actigraphy analyzed with 6 different algorithms and polysomnography (PSG) measurement of Wake after sleep onset (WASO). Axes are in minutes. None of the algorithms were significantly related to PSG WASO. With regression slope (red) and 95% confidence interval (shaded blue).

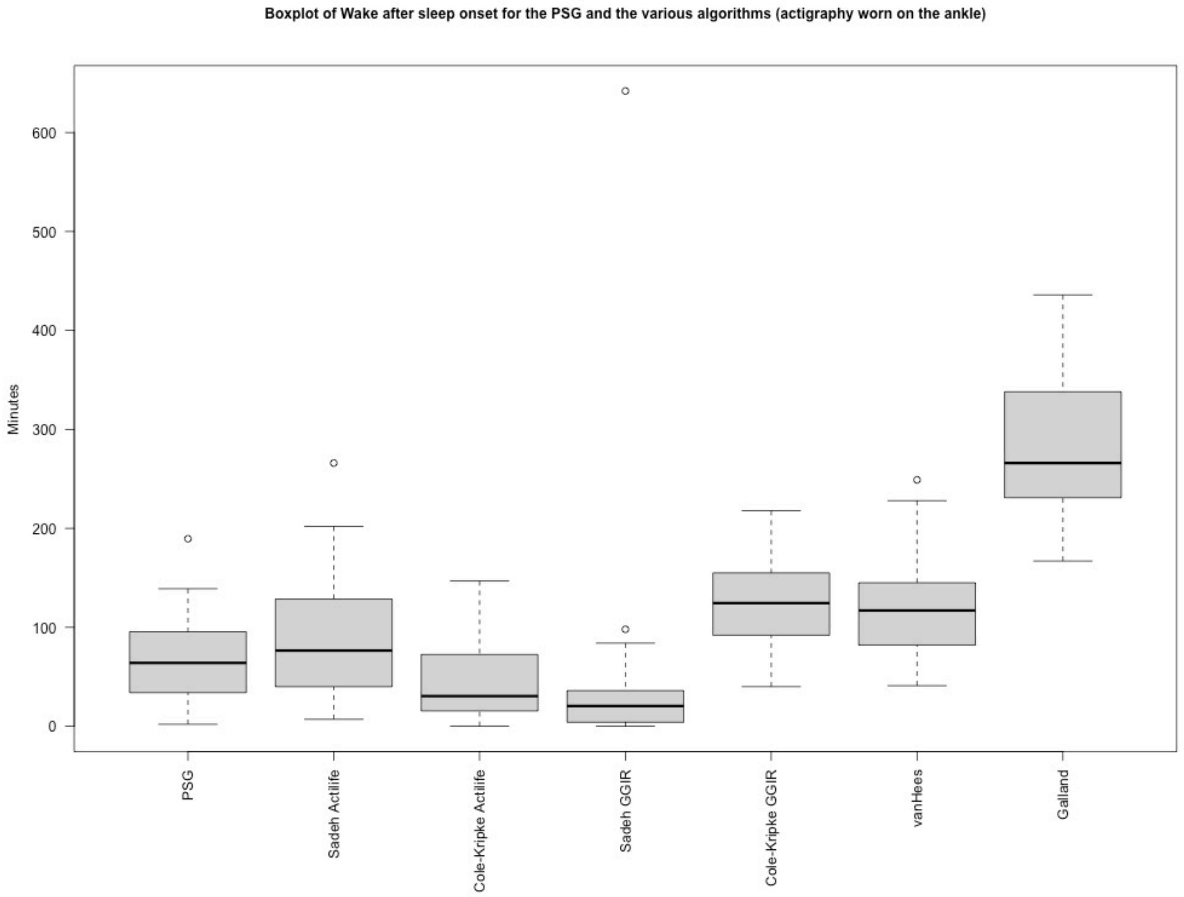

Figure 10 Boxplot (median, first and third quartiles, and range) of Wake after sleep onset measured from ankle actigraphy and PSG.

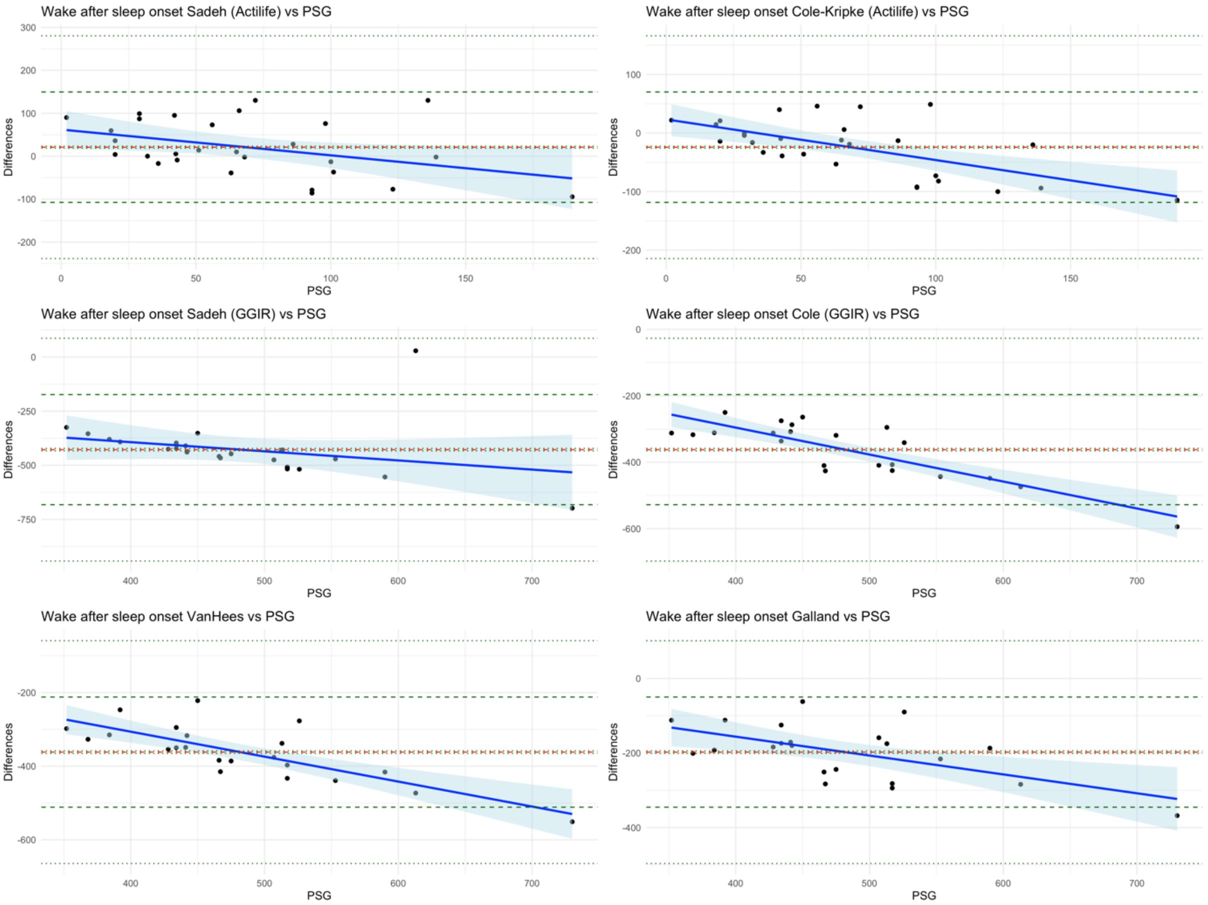

Figure 11 Bland-Altman plots of ankle-worn actigraphy analyzed with 6 different algorithms and polysomnography (PSG) measurement of Wake after sleep onset on the same night. Axes are in minutes. Cole-Kripke (Actilife, r= -0.69 and GGIR, r = -0.81), and vanHees (r= -0.67), showed proportional bias. With regression slope (blue ----) and 95% confidence interval (shaded blue), mean difference (red - - -), and upper and lower limits of agreement (green - - -).

S4. Analysis sleep efficiency

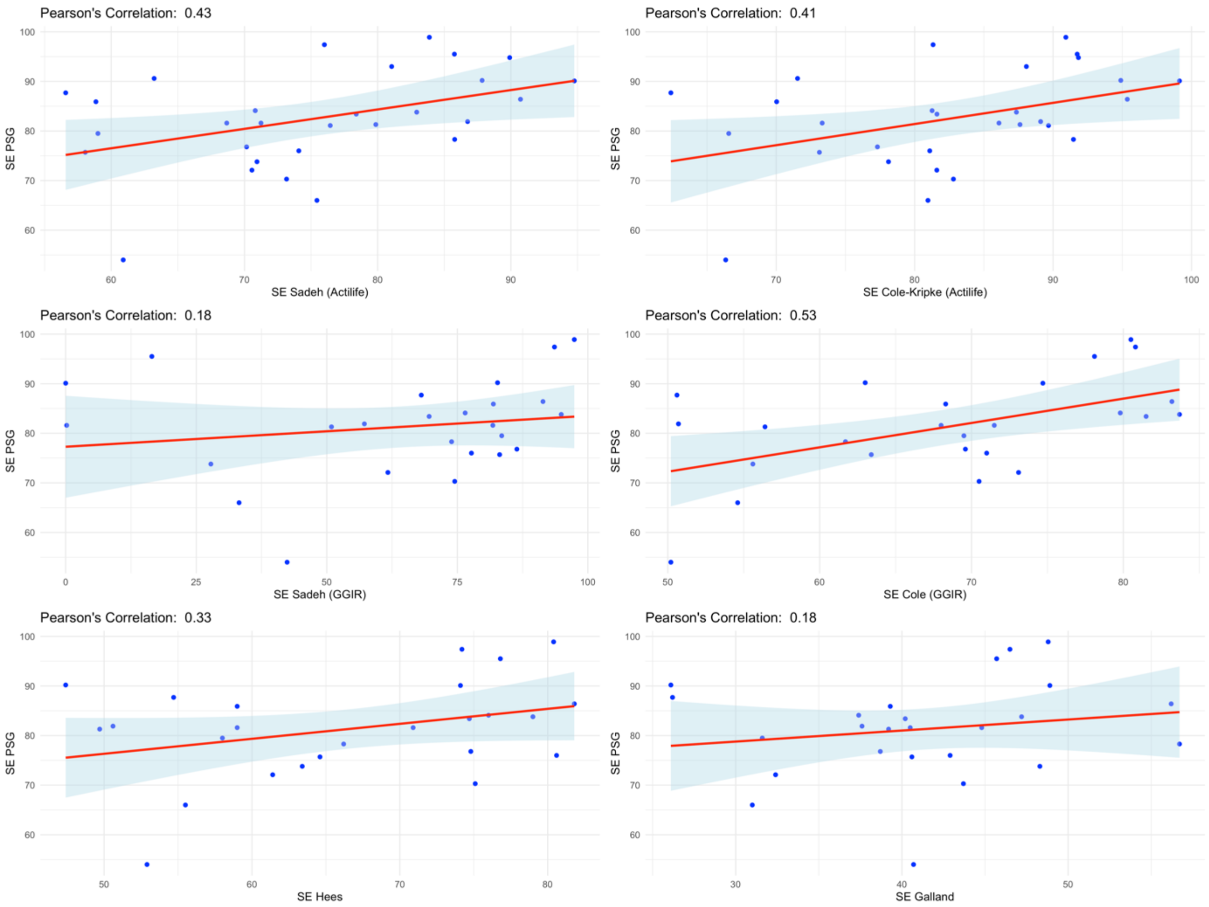

Figure 12 Correlation of wrist-worn actigraphy analyzed with 6 different algorithms and polysomnography (PSG) measurement of Sleep efficiency. Axes are in percentages. None of the algorithms were significantly related to PSG sleep efficiency. With regression slope (red) and 95% confidence interval (shaded blue).

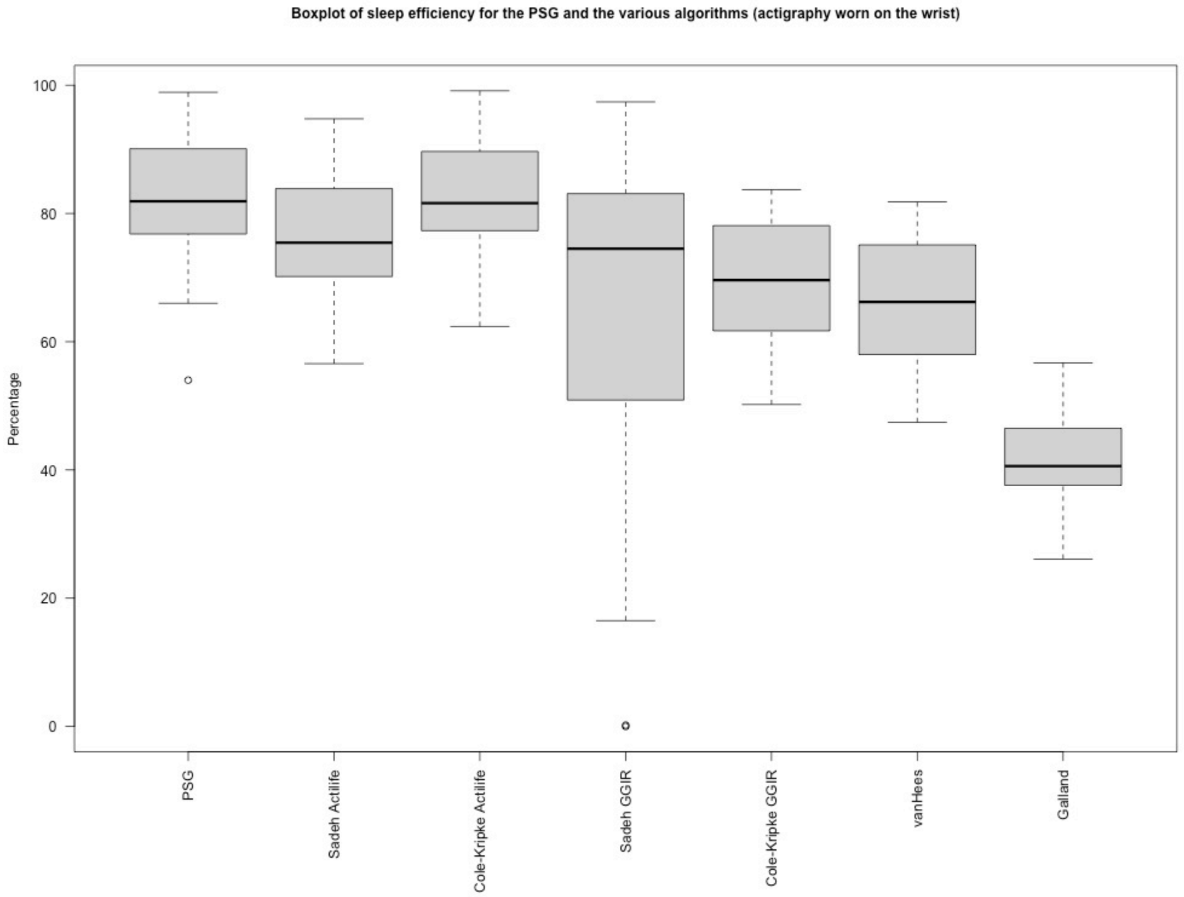

Figure 13 Boxplot (median, first and third quartiles, and range) of Sleep efficiency measured from wrist actigraphy and PSG.

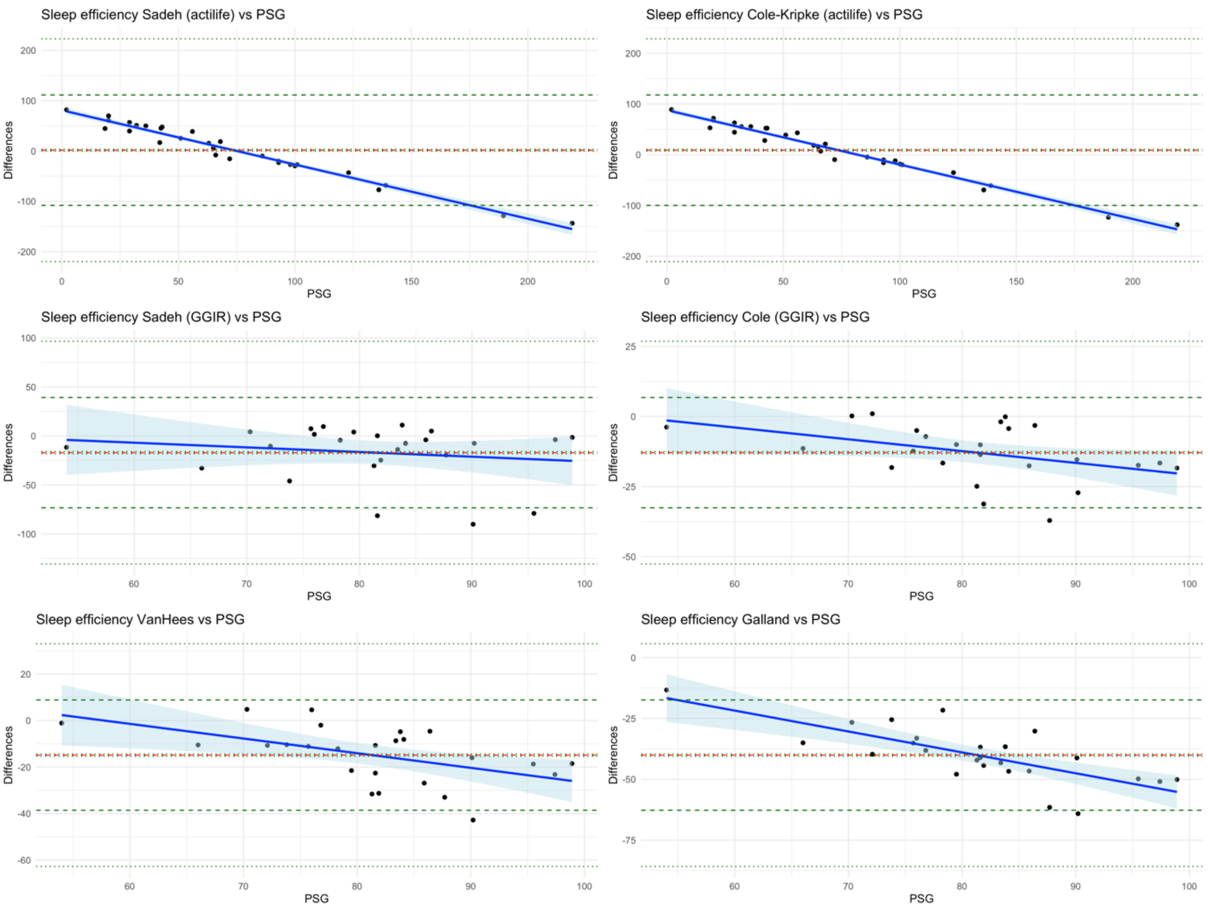

Figure 14 Bland-Altman plots of wrist-worn actigraphy analyzed with 6 different algorithms and polysomnography (PSG) measurement of sleep efficiency on the same night. Axes are in percentages. Sadeh (Actilife, r = -1.07), Cole-Kripke (Actilife, r = -1.07) and Galland (r = -0.85) showed significant proportional bias . With regression slope (blue ----) and 95% confidence interval (shaded blue), mean difference (red - - -), and upper and lower limits of agreement (green - - -).

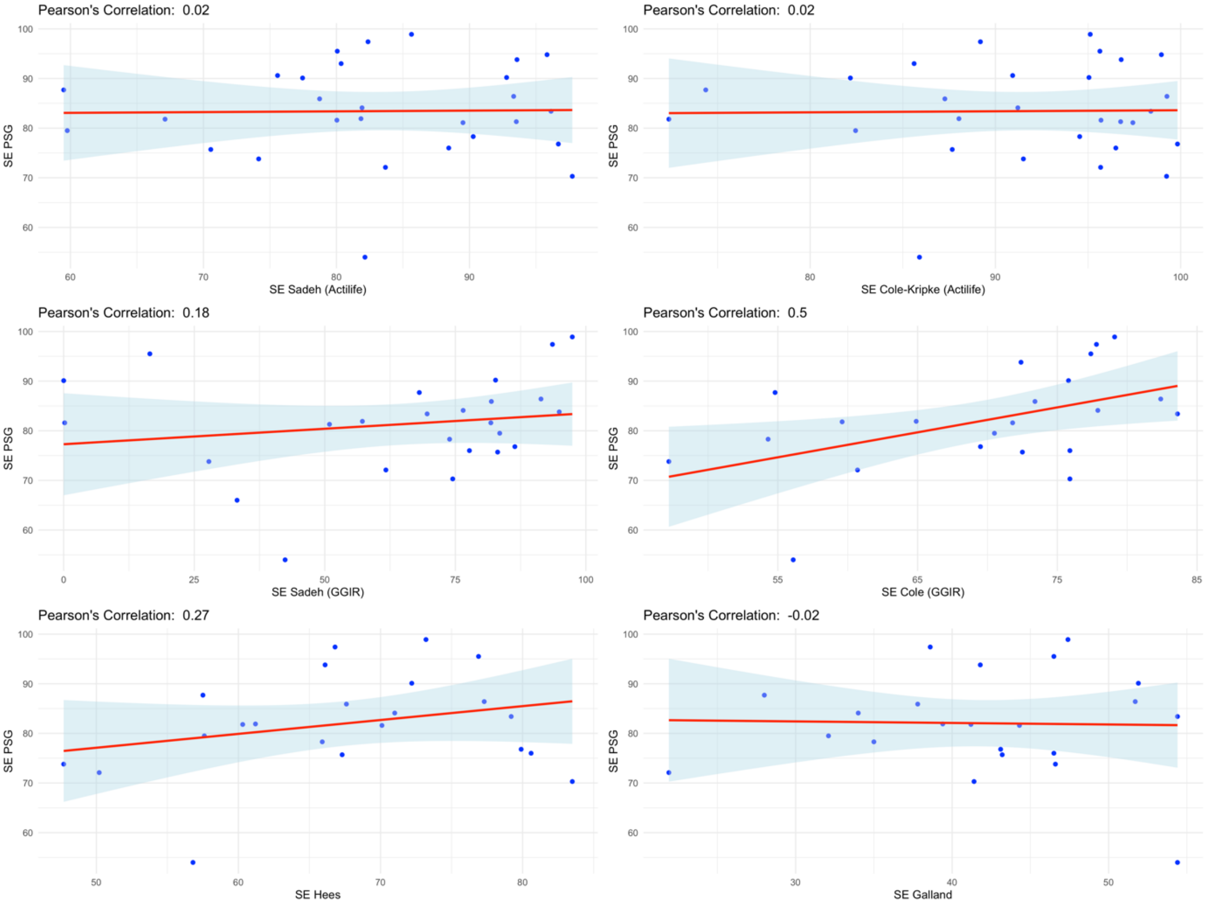

Figure 15 Correlation of ankle-worn actigraphy analyzed with 6 different algorithms and polysomnography (PSG) measurement of Sleep efficiency. Axes are in percentages. None of the algorithms were significantly related to PSG Sleep efficiency. With regression slope (red) and 95% confidence interval (shaded blue).

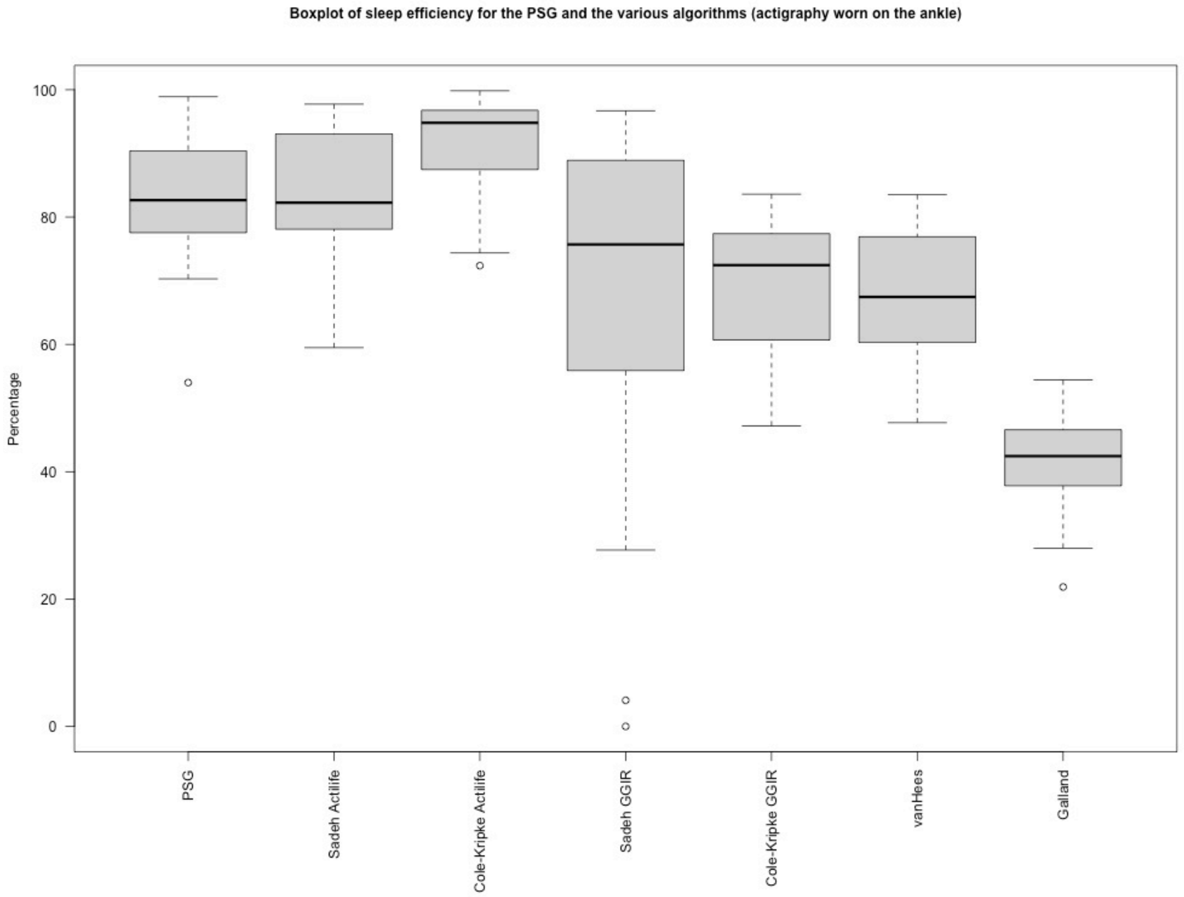

Figure 16 Boxplot (median, first and third quartiles, and range) of Sleep efficiency measured from ankle actigraphy and PSG.

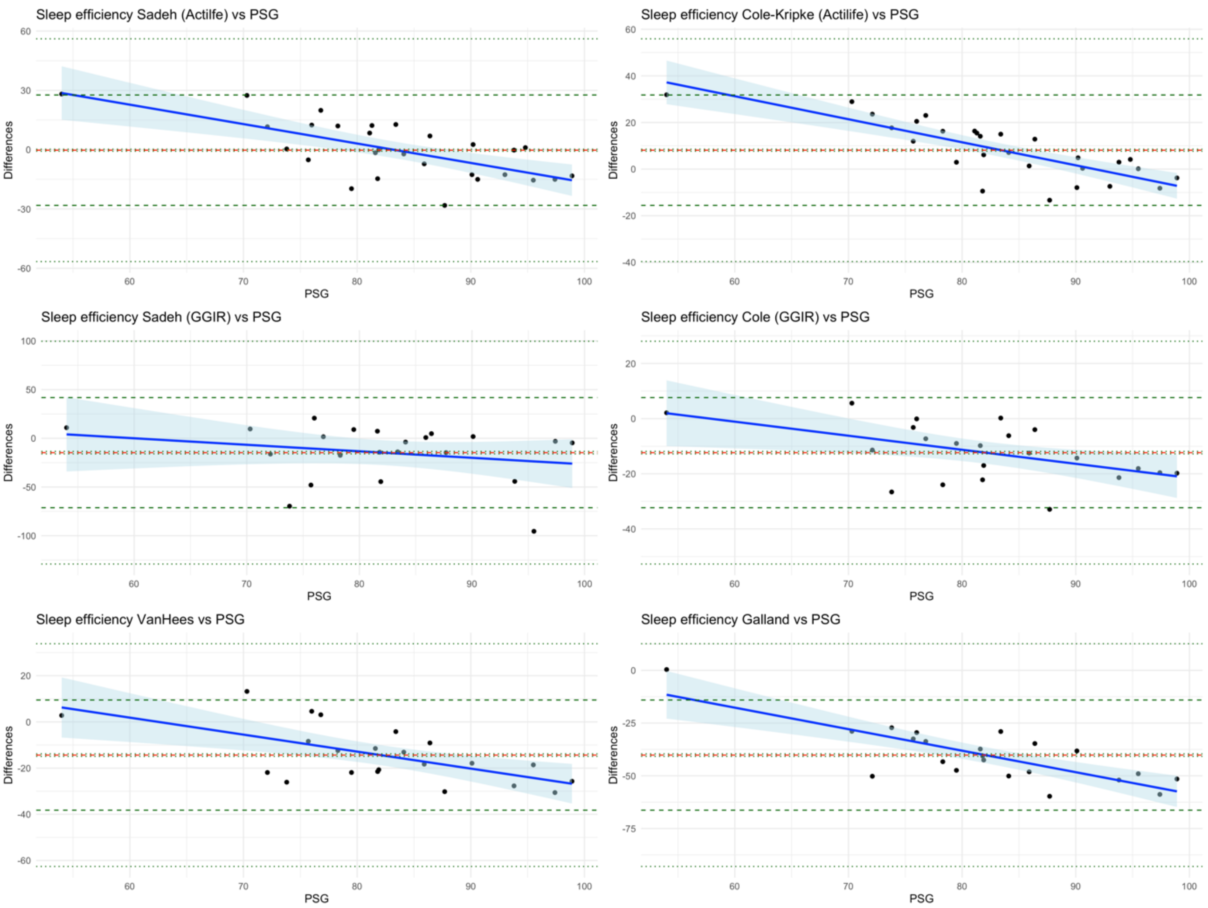

Figure 17 Bland-Altman plots of ankle-worn actigraphy analyzed with 6 different algorithms and polysomnography (PSG) measurement of sleep efficiency on the same night. Axes are in percentages. Sadeh (Actilife, r = -1.0), Cole-Kripke (Actilife, r = -1.0) and Galland (r = -1.02) showed significant proportional bias. With regression slope (blue ----) and 95% confidence interval (shaded blue), mean difference (red - - -), and upper and lower limits of agreement (green - - -).

S5. Analysis awakenings

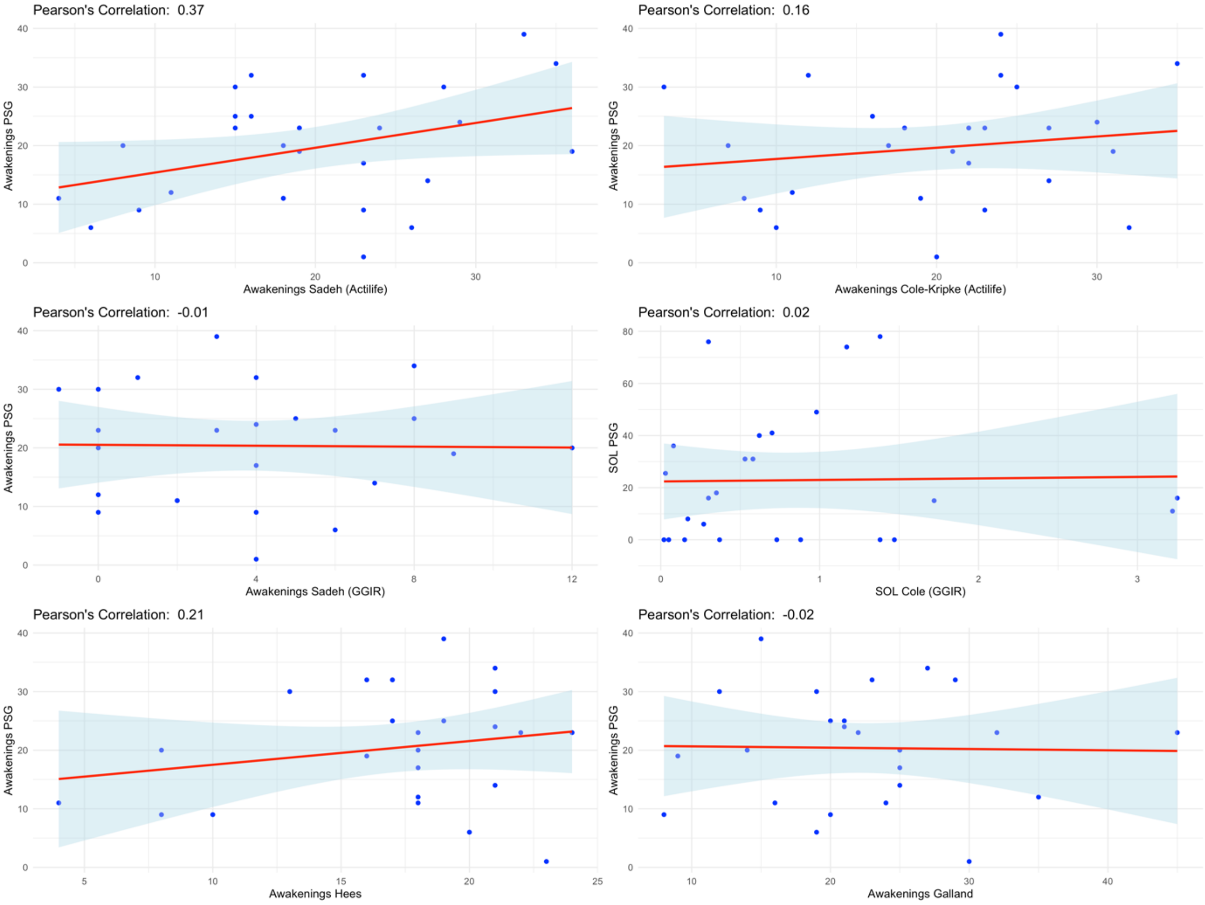

Figure 18 Correlation of wrist-worn actigraphy analyzed with 6 different algorithms and polysomnography (PSG) measurement of Awakenings. Axes are counts. None of the algorithms were significantly related to PSG Awakenings. With regression slope (red) and 95% confidence interval (shaded blue).

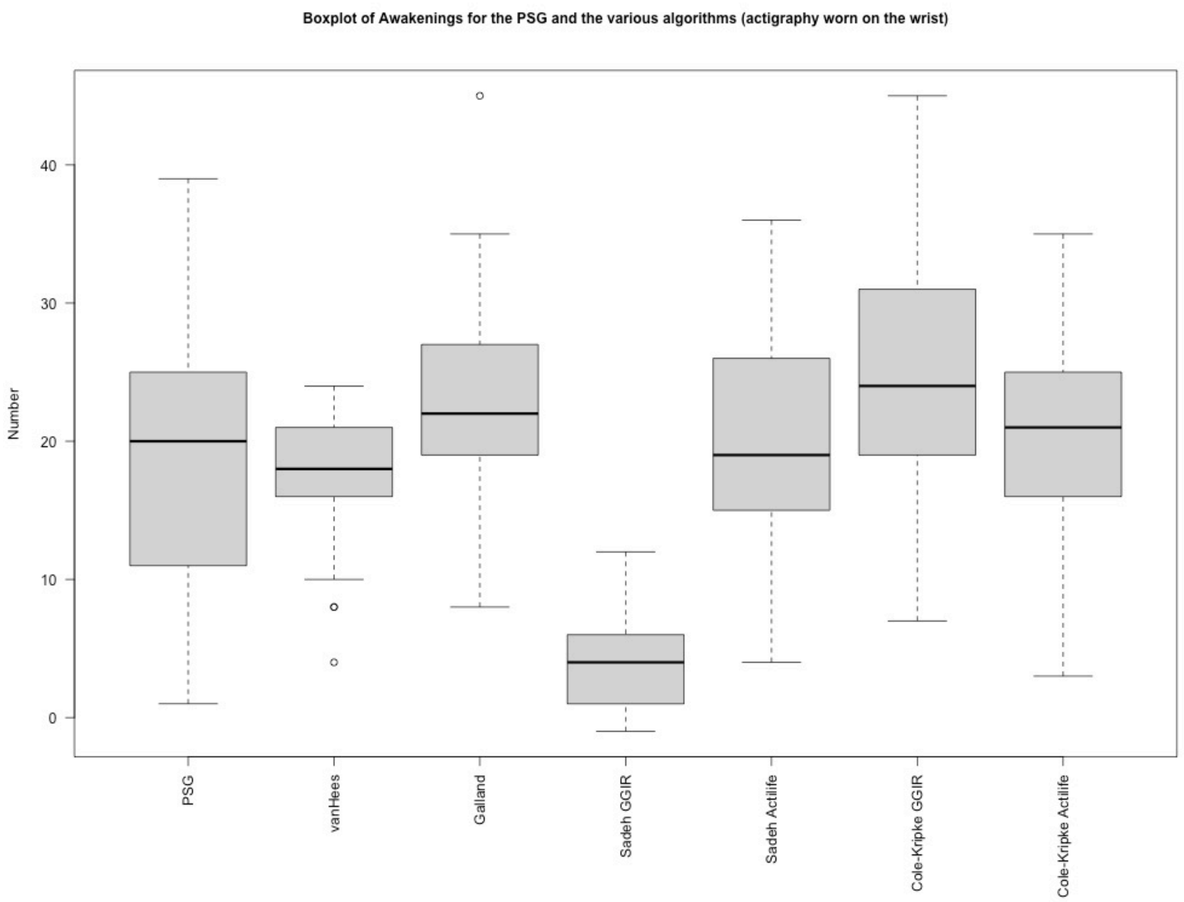

Figure 19 Boxplot (median, first and third quartiles, and range) of Sleep efficiency measured from wrist actigraphy and PSG.

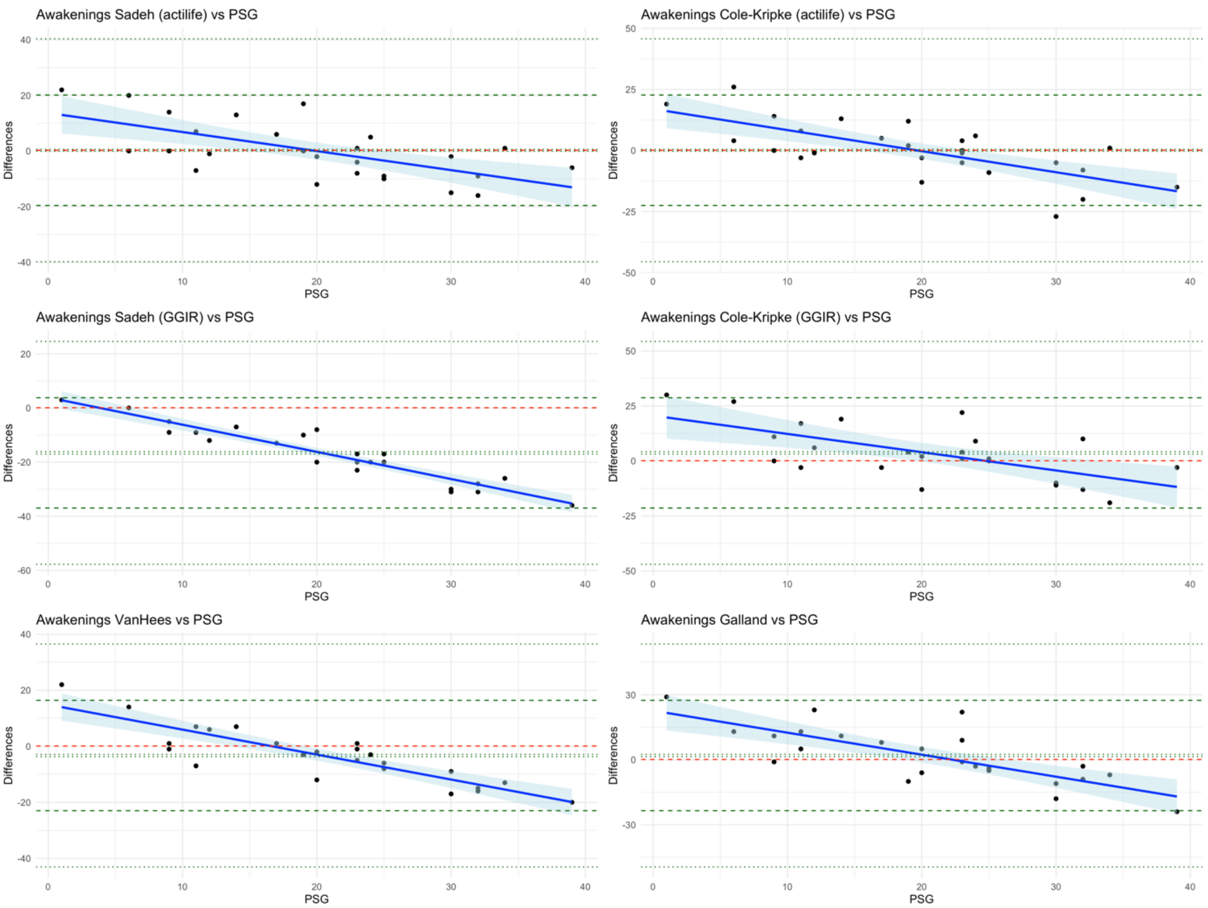

Figure 20 Bland-Altman plots of wrist-worn actigraphy analyzed with 6 different algorithms and polysomnography (PSG) measurement of awakenings on the same night. Axes are counts. All algorithms showed significant proportional bias (r = approximately -1.0). With regression slope (blue ----) and 95% confidence interval (shaded blue), mean difference (red - - -), and upper and lower limits of agreement (green - - -).

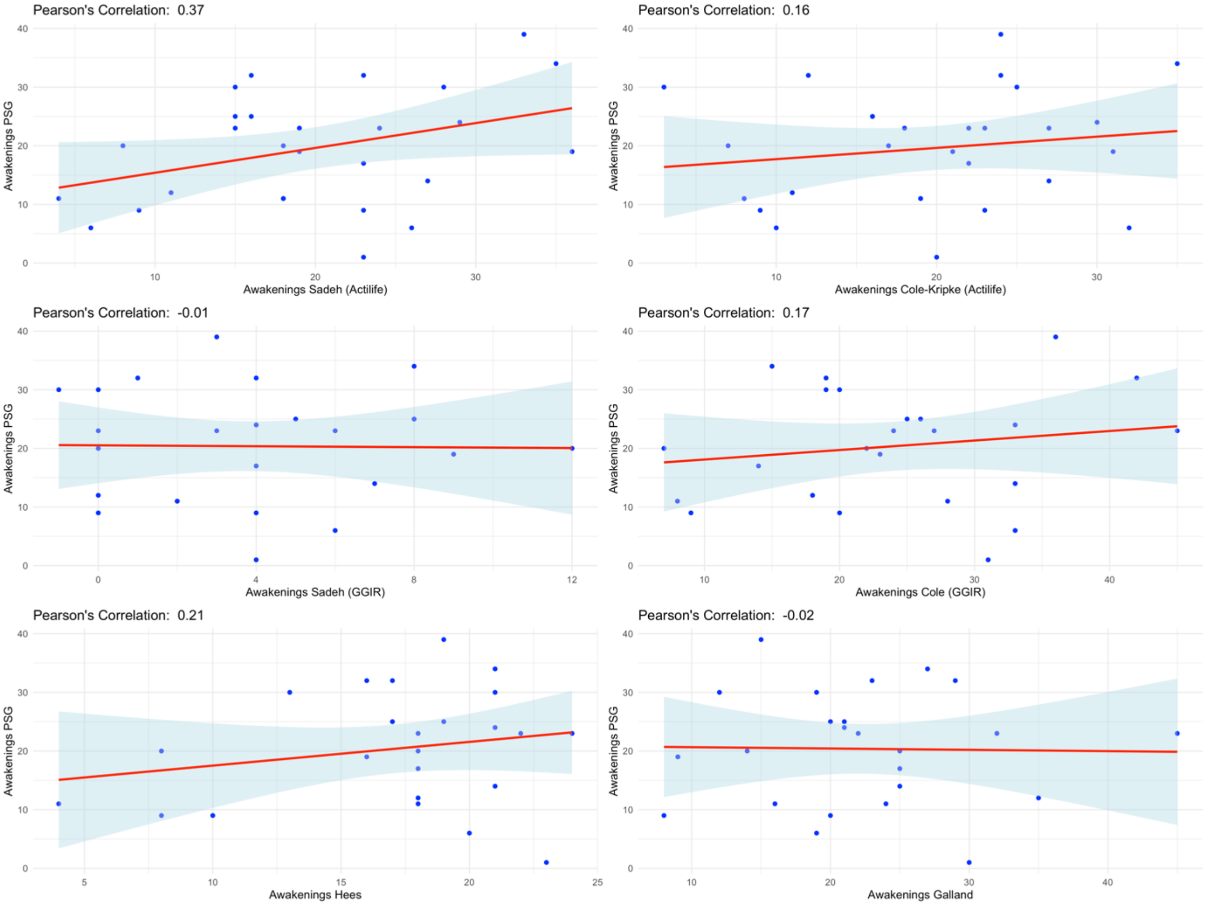

Figure 21 Correlation of ankle-worn actigraphy analyzed with 6 different algorithms and polysomnography (PSG) measurement of Awakenings. Axes are counts. None of the algorithms were significantly related to PSG Awakenings. With regression slope (red) and 95% confidence interval (shaded blue).

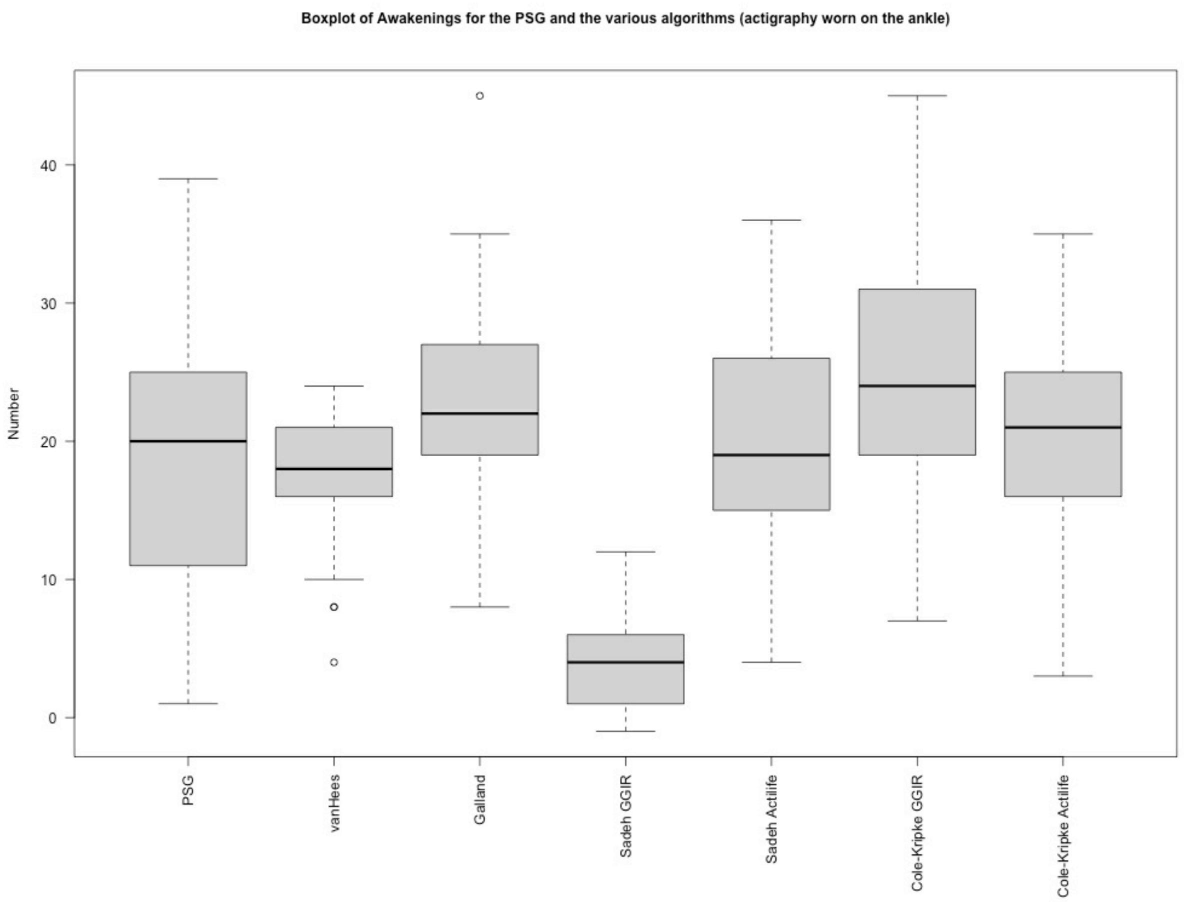

Figure 22 Boxplot (median, first and third quartiles, and range) of Sleep efficiency measured from ankle actigraphy and PSG.

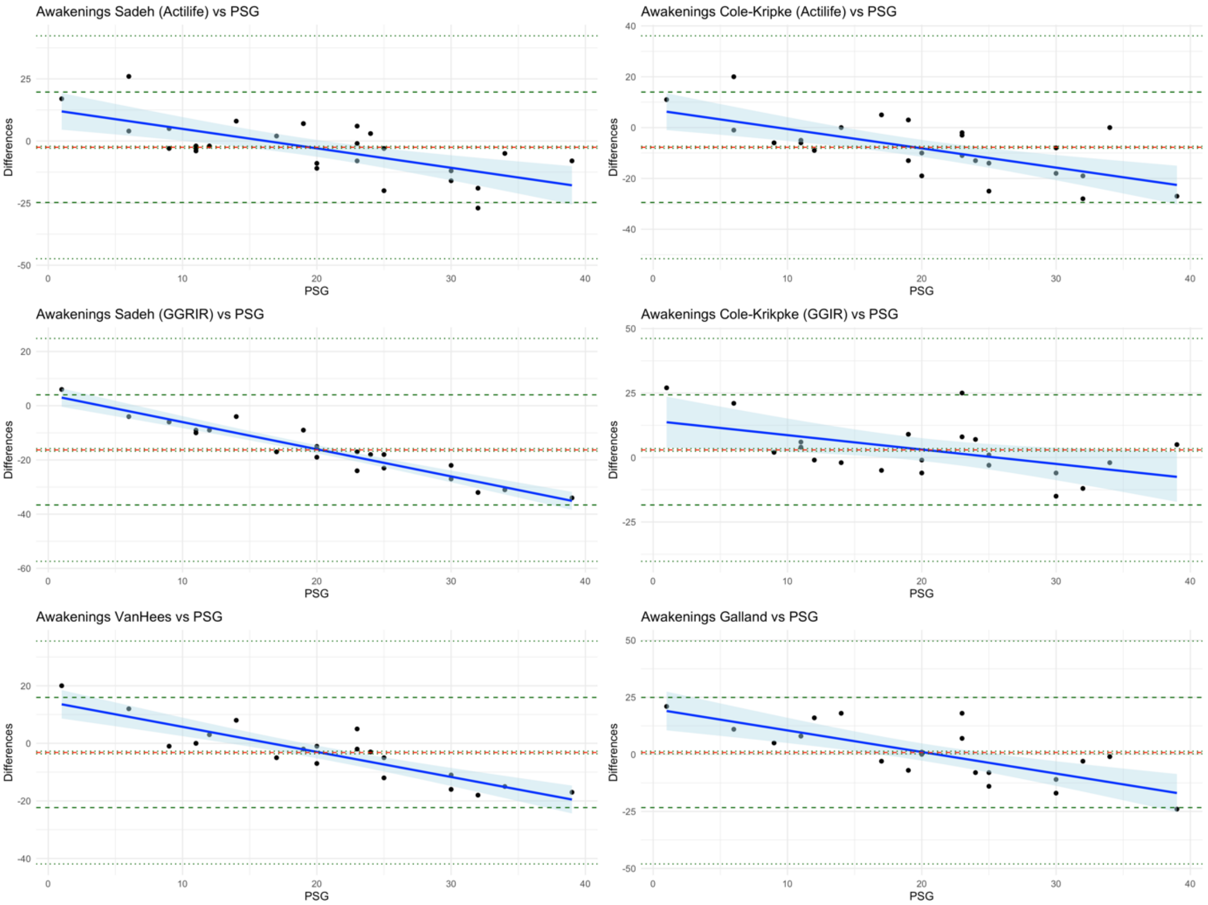

Figure 23 Bland-Altman plots of ankle-worn actigraphy analyzed with 6 different algorithms and polysomnography (PSG) measurement of awakenings on the same night. Axes are counts. All algorithms, except Cole-Kripke (GGIR) showed significant proportional bias (r = approximately -1.0). With regression slope (blue ----) and 95% confidence interval (shaded blue), mean difference (red - - -), and upper and lower limits of agreement (green - - -).
